# Porous microneedle patch with sustained delivery of extracellular vesicles mitigates severe spinal cord injury

**DOI:** 10.1038/s41467-023-39745-2

**Published:** 2023-07-07

**Authors:** Ao Fang, Yifan Wang, Naiyu Guan, Yanming Zuo, Lingmin Lin, Binjie Guo, Aisheng Mo, Yile Wu, Xurong Lin, Wanxiong Cai, Xiangfeng Chen, Jingjia Ye, Zeinab Abdelrahman, Xiaodan Li, Hanyu Zheng, Zhonghan Wu, Shuang Jin, Kan Xu, Yan Huang, Xiaosong Gu, Bin Yu, Xuhua Wang

**Affiliations:** 1 Department of Rehabilitation Medicine of First Affiliated Hospital and School of Brain Science and Brain Medicine, Zhejiang University School of Medicine, 310003 Hangzhou, Zhejiang Province P. R. China; 2 Liangzhu Laboratory, MOE Frontier Science Center for Brain Science and Brain-machine Integration, State Key Laboratory of Brain-machine Intelligence, Zhejiang University, 1369 West Wenyi Road, 311121 Hangzhou, China; 3 NHC and CAMS Key Laboratory of Medical Neurobiology, Zhejiang University, 310058 Hangzhou, China; 4 Department of Orthopedics of 2nd Affiliated Hospital and School of Brain Science and Brain Medicine, Zhejiang University School of Medicine, Zhejiang University, 310003 Hangzhou, Zhejiang Province, PR China; 5 Department of Hepatobiliary and Pancreatic Surgery, Affiliated Hospital of Nantong University, Medical School of Nantong University, 226001 Nantong, China; 6 Key Laboratory of Neuroregeneration of Jiangsu and Ministry of Education, NMPA Key Laboratory for Research and Evaluation of Tissue Engineering Technology Products, Nantong University, Nantong, China; 7 Co-innovation Center of Neuroregeneration, Nantong University, 226001 Nantong, Jiangsu P. R. China

**Keywords:** Spinal cord injury, Biomedical materials, Mesenchymal stem cells, Biomedical engineering, Biomaterials - cells

## Abstract

The transplantation of mesenchymal stem cells-derived secretome, particularly extracellular vesicles is a promising therapy to suppress spinal cord injury-triggered neuroinflammation. However, efficient delivery of extracellular vesicles to the injured spinal cord, with minimal damage, remains a challenge. Here we present a device for the delivery of extracellular vesicles to treat spinal cord injury. We show that the device incorporating mesenchymal stem cells and porous microneedles enables the delivery of extracellular vesicles. We demonstrate that topical application to the spinal cord lesion beneath the spinal dura, does not damage the lesion. We evaluate the efficacy of our device in a contusive spinal cord injury model and find that it reduces the cavity and scar tissue formation, promotes angiogenesis, and improves survival of nearby tissues and axons. Importantly, the sustained delivery of extracellular vesicles for at least 7 days results in significant functional recovery. Thus, our device provides an efficient and sustained extracellular vesicles delivery platform for spinal cord injury treatment.

## Abstract (web summary)

Efficient delivery of extracellular vesicles to the injured spinal cord, with minimal damage, remains challenging. Here, the authors fabricate a minimally invasive microneedle device, which provides efficient and sustained extracellular vesicle delivery for spinal cord injury treatment.

## Introduction

There are approximately 930,000 new spinal cord injury (SCI) cases caused by accidents each year^1^. Even though the systemic administration of methylprednisolone sodium succinate (MPSS) has been approved as the only drug for treating acute SCI, this drug has limited therapeutic efficacy and severe side effects, which severely limits its application in clinical practice^2,3^. SCI is initially caused by mechanical trauma and diverse mechanisms of secondary damage following the inflammatory response^4^. Secondary damage can cause hemorrhage, edema, decreased blood flow and inflammation, thus affecting adjacent spinal cord segments^5^. The inflammatory environment triggered by the activation of microglia and the release of proinflammatory cytokines leads to inevitable neuronal damage and, consequently, scar and cystic cavity formation in spinal cord lesion sites^6,7^. Recently, extensive studies have demonstrated that mesenchymal stem cells (MSCs) delivered intravenously (IV) promote functional recovery after contusive SCI^8–11^. However, the lung has the ability to clear over 60% of the administered MSCs, resulting in a decrease in the number of MSCs that can be home to the lesion site to the injured site and play a therapeutic role^12^. Although some cells in the lungs might survive for 2–3 days and release exosomes which can potentially travel to the target injury site, the patients may experience coughing side effects^13,14^. Alternatively, the transplantation of MSCs directly into the lesion site has been proposed as an effective treatment to alleviate neuroinflammation triggered by SCI^15^. However, the neuroinflammatory microenvironments of spinal cord injury (SCI) lesions have been shown to be detrimental to the survival and function of mesenchymal stem cells (MSCs), thus posing a significant obstacle to the clinical application of these cells^11^.

Fortunately, it has been reported that MSC secretome, particularly extracellular vesicles (MSC-EVs), offer similar therapeutic benefits for immune modulation in animal models of SCI^16–18^. Previous studies have shown that MSC-EVs immobilized in a peptide-modified adhesive hydrogel transplanted into an SCI lesion during the acute phase could mitigate the SCI microenvironment and promote functional recovery^19–21^. However, the retention time of MSC-EVs in spinal tissue by directly transplantation might not be long enough for them to exhibit their optimal therapeutic effects^17,22^. The neuroinflammation and neuronal apoptosis caused by SCI could continue for a couple of weeks or even longer^4^, but MSC-EVs could not be continuously delivered to SCI lesions by these methods. Moreover, direct transplantation of MSCs or MSC-EVs into the spinal cord injury site may result in damage to healthy tissues adjacent to the lesion, potentially leading to unpredictable side effects^23^. Therefore, direct transplantation of materials containing MSCs or MSC-EVs into the spinal cord lesion of an acute SCI patient is not an optimized approach.

**Fig. 1:**
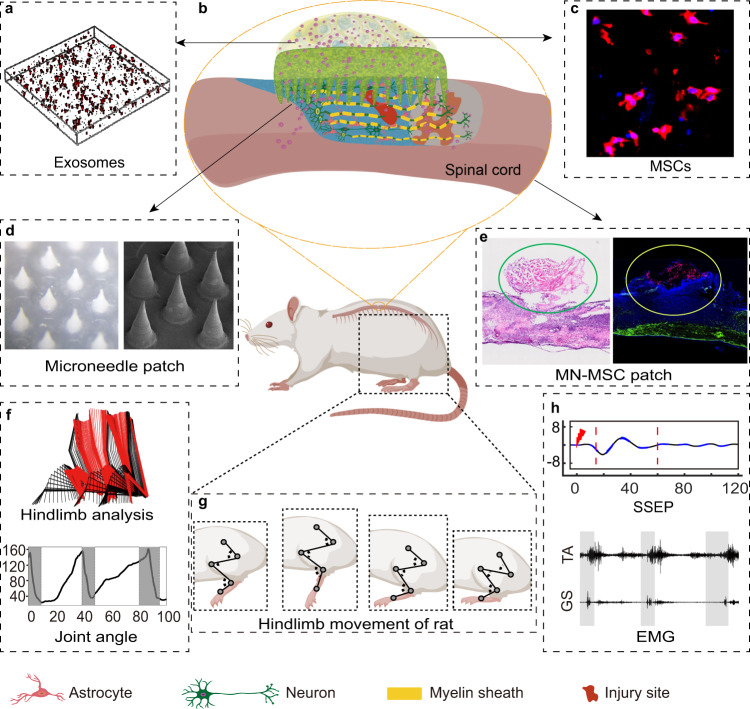
Schematic illustration of MN-MSC patch implantation at the site of the spinal cord injury. **a** Schematic depicting exosomes embedded in GelMA hydrogel. **b** Schematic of the MN-MSC patch. **c** Representative microscopy images showing MSCs in the patch. **d** Representative morphological images of the Microneedle patch. **e** Representative microscopy images of the MN-MSC patch applied to the spinal cord. **f** Analysis of hindlimb function including joint angle. **g** Illustration of hindlimb movement. **h** Electrophysiological analysis of hindlimb movement.

To overcome these challenges, we took advantage of a microneedle (MN) array, which has facilitated painless localized delivery of drugs or therapeutic biomolecules with good tolerability in clinical trials^24–26^. We then fabricated a patch with an MN array with appropriate mechanical strength matching soft spinal tissues and with a suitable pore size for MSC-EV delivery when it was mounted onto a spinal cord lesion beneath the dura^27,28^. To sustainably deliver MSC-EVs, the MN arrays were additionally mounted with a gelatin methacryloyl (GelMA) hydrogel block embedded with MSCs during surgery (Fig. 1). We envisioned that the GelMA hydrogel block could provide a biocompatible microenvironment for promoting the long-term survival of MSCs. In this way, we achieved a sustained delivery of MSC secretome to the spinal cord lesion via the use of porous MNs. This approach was proposed to enhance therapeutic efficacy without requiring direct invasion of MSCs into the spinal cord (Fig. 1). We showed that MN-MSC patch can effectively maintain the survival of MSCs and sustainably release MSC secretome for at least one week, which led to remarkable neuroprotection effects, superior muscle control, and robust functional recovery following severe SCI (Fig. 1).

## Results

### Fabrication and characterization of the patch with an MN array In Vitro

**Fig. 2:**
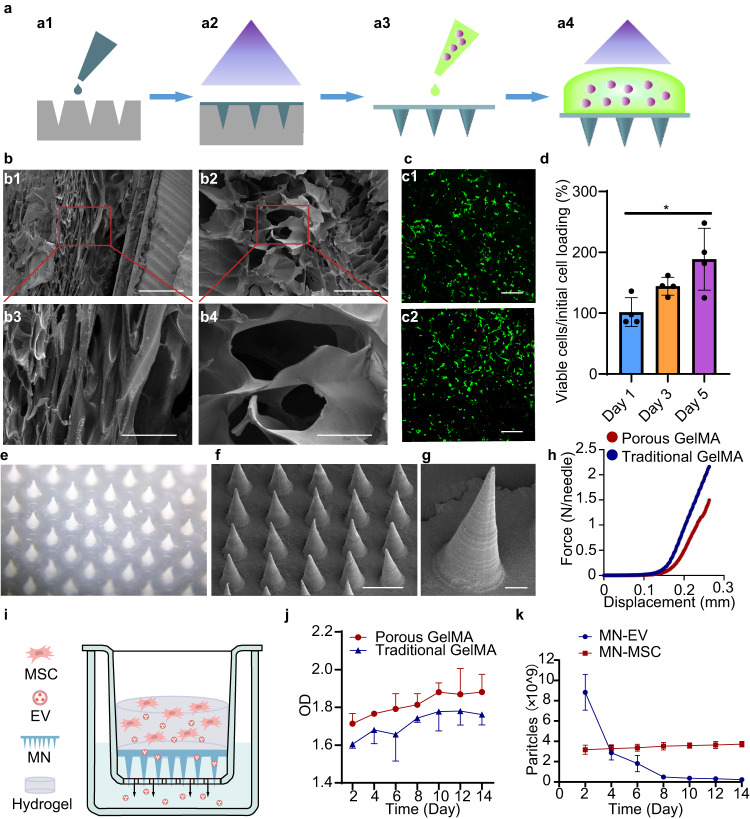
Fabrication and characterization of the MN-MSC patch. **a** Schematic of the MN-MSC patch fabrication process by a1) casting, a2) blue light crosslinking, a3) peeling from the PDMS mold and adding GelMA solution with MSCs, and a4) blue light crosslinking. **b** SEM image of the b1) interstructure of traditional and b2) porous GelMA hydrogels. Scale bars of images (b1, b2) indicate 300 µm, and scale bars of images (b3, b4) indicate 100 µm. The experiment was repeated 3 times independently with similar results. **c** Calcein AM (live)/EthD (dead) staining revealed the morphology and viability of MSCs encapsulated in GelMA hydrogel on c1) day 3 and c2) day 5. Scale bars: 200 μm. **d** Quantitative viability analysis of MSCs encapsulated in GelMA hydrogel on days 1, 3 and 5. Data are presented as the means ± SEM (*n* = 4 independent GelMA hydrogels with MSCs). Statistical analysis was performed using one-way ANOVA followed by Tukey’s multiple comparisons test and two-tailed paired *t* tests were used for comparisons between two groups. Total: *F* = 6.808, *p* = 0.0158. Day 1 vs Day 3, *p* = 0.2211; Day 1 vs Day 5, *p* = 0.0125; Day 3 vs Day 5, *p* = 0.1994. * indicates *p* < 0.05. **e** Representative optical microscopy images and (**f**, **g**) SEM images of MNs. Scale bars for images (**f**) indicate 500 µm, and scale bars for images (**g**) indicate 50 µm. The experiment was repeated 3 times independently with similar results. **h** The mechanical strength of MNs. **i** Schematic showing the study design used to test the release profile of exosomes from the MN-MSC patch. **j** The daily proteins release curves of MN-MSC patches fabricated with porous and normal GelMA hydrogels. Data are presented as the means ± SEM (*n* = 3 independent patches for each group). **k** The daily exosome release curves of MN-MSC and MN-EV patches. Data are presented as the means ± SEM (*n* = 3 independent patches for each group).

To characterize the produced MSCs, we conducted flow cytometry analysis. According to well-accepted criteria for the identification of specific cell-surface markers (that is, CD73^+^, CD90^+^, CD105^+^, CD45^−^, CD34^−^, CD19^−^, CD14^−^, CD11b^−^, CD79a^−^, and HLA-DR)^9^, the harvested cells were identified as MSCs (Fig. S1), suggesting the high quality of MSCs used in our study. To achieve sustainable release of MSC secretome, we designed a patch containing an MN array (Fig. 2a). The MN patch was made from chemically cross-linked methacryloyl (MW. 250 kDa) with a micromolding method to form GelMA (Fig. 2a, a1, a2)^29,30^. The scale of the fabricated MN patch was approximately 4 mm × 4 mm with 45 needles, which is large enough to cover a typical rat contusive spinal cord lesion (Fig. S3). The needle of the MN arrays was conical, with a base diameter of 250 μm, a height of 600 μm and a tip diameter of 10 μm (Fig. 2e–g). To sustainably release extracellular vesicles, the patch was additionally covered with GelMA hydrogel embedded with MSCs during surgery (Fig. S3f–i). The GelMA hydrogel with porous structures was designed to facilitate the release of MSC-EVs through the polymeric needles. As shown in the scanning electron microscopy (SEM) images, the pore size of the gel reached up to approximately 100 μm (Fig. 2b, b2, b4), compared with the relatively small pore size of 10 μm in the traditional GelMA hydrogel (Fig. 2b, b1, b3).

To examine the biocompatibility of the MN patch, we first dropped the methacryloyl solution with MSCs (40 μl, approximately 2.5 × 10^7^ cells/mL) onto the surface of the patch and cross-linked methacryloyl to form GelMA hydrogel with blue light irradiation (Fig. 2a, a3, a4). The live/dead staining of the GelMA hydrogel revealed excellent viability of embedded MSCs after culturing for 3 and 5 days (Fig. 2c). Quantitative analysis with the CCK-8 test indicated that MSCs in the GelMA hydrogel of the MN-MSC patch survived and proliferated well (Fig. 2d). The needle with a porous structure exhibited comparatively lower mechanical strength than the conventional needle (porous 10.77 kPa vs traditional 30.92 kPa, Fig. S3e), and matched that of the soft rat’s spinal cord tissues (around 8.1 kPa)^27,28^. We next assessed the MSC-EV release capacity of the MN patch with this porous structure. To do so, the patches made from porous GelMA^26^ or regular GelMA were seeded with MSCs and placed in the upper compartment of a Transwell (Fig. 2i). The Transwells were then immersed in culture media and incubated at 37 °C. At regular intervals, aliquots of samples were collected from the bottom wells, and the concentrations of MSC-secretomes in the samples were measured by a Micro BCA Protein Assay kit^31^. Plotting the optical density (OD) of the samples revealed that the MN patch made from porous GelMA released MSC-secretome more efficiently (Fig. 2j), possibly because the MN patch with a larger pore size was more permeable for MSC-secretome. We therefore chose the MN patch made from porous GelMA for the following studies.

Previous studies have demonstrated that extracellular vesicles (EVs) derived from stem cells show promise as a potential alternative to stem cell therapy^16–18^, we therefore sought to evaluate the sustained MSC-EVs delivery capacity of MN patches embedded with MSCs (MN-MSC). The MN patches directly loaded with MSC-EVs (MN-EV) were used as controls. The MSC-EVs were isolated from MSC culture supernatant via ultracentrifugation and showed a typical cup-shaped morphology (Fig. S2a). Through nanoparticle tracking analysis (NTA), we found that the MSC-EVs had an average size of 110 nm, which was consistent with the results of previous studies (Fig. S2c)^20,32^. The expression of tetrapanins CD9 and TSG101 (mesenchymal markers) on the collected samples (Fig. S2d) indicated successful isolation and purification of MSC-EVs. We then compared the persistent MSC-EV release capacity of MSC-seeded and MSC-EV-loaded MN patches. The numbers of MSC-EVs released in the collected samples from the lower wells of the Transwell system at various time points were plotted (Fig. 2k). The results indicated that the release of MSC-EVs from MSC-EV-loaded MN patches initially declined rapidly within the first 4 days, whereas the delivery of MSC-EVs from MSC-seeded MN patches remained stable, with a slight increase at 2 weeks (Fig. 2k). This suggests that the MN patches exhibited excellent biocompatibility, allowing for the survival of MSCs. Thus, we successfully fabricated a device for the sustained and efficient delivery of MSC-EVs.

### Distribution of EVs in the injured spinal cord after MN patch implantation

**Fig. 3:**
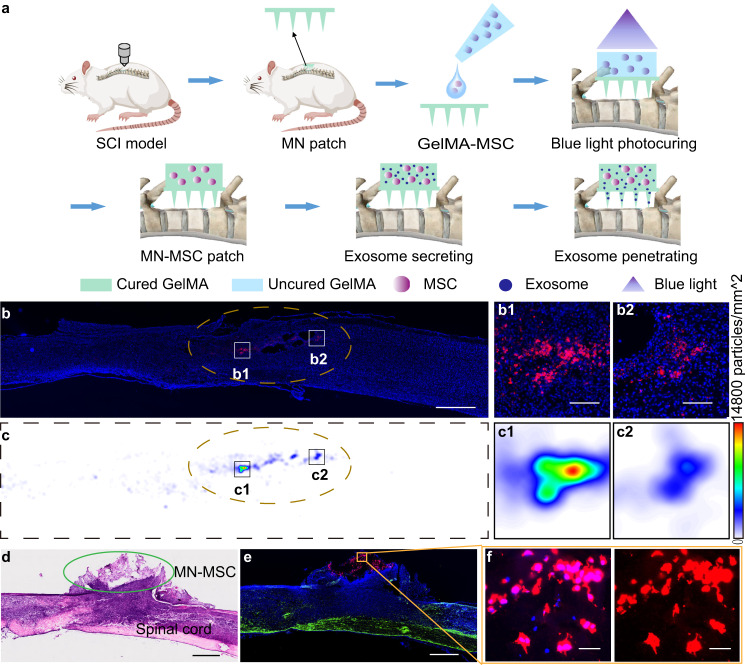
Sustained MSC-EV delivery by MN-MSC patch treatment alleviates neuroinflammation triggered by SCI. **a** Schematic illustration of MN-MSC patch implantation on the injury site of the spinal cord. **b** In vivo distribution of exosomes (DiI labeled, red) in the injured spinal cord tissues after MN-EV patch implantation, blue (DAPI). Scale bar: 1 mm. The injury region of the spinal cord is marked with yellow dotted lines. **c** Heatmaps of exosome distribution in the injured spinal cord tissues after MN-EV patch implantation; red: the highest numbers of exosomes, blue: the lowest, and white: background. The injury region of the spinal cord is marked with yellow dotted lines. b1, b2, c1, c2) Enlarged images to show the details. Scale bar: 100 μm. **d** HE staining of the injured spinal cord with MN-MSC patch implantation; scale bar: 1 mm. The experiment was repeated 3 times independently with similar results. The experiment was repeated 3 times independently with similar results. **e** Representative images of immunohistochemical staining for MN-MSC patches on the injured spinal cord on day 7 after SCI. Green (GFAP), blue (DAPI), red (GAPDH), scale bar: 1 mm. The experiment was repeated 3 times independently with similar results. **f** High-magnification images of the boxed area, scale bar: 50 µm. The experiment was repeated 3 times independently with similar results.

After verifying that the fabricated MN-MSC patch could sustainably deliver EVs in vitro, we sought to assess whether the MSC-EVs of the patch could be delivered through the MNs into injured spinal cord tissues in a contusive rat SCI model. We used a severe T10 contusive SCI model, which was constructed by an infinite vertical impactor with the parameters of a 2.5 m/s rate, 2 mm depth, 5 s duration time and 3 mm diameter cylinder^33^. Because MSC-EVs in live MSCs are difficult to label, MSC-EVs stained with CM-DiI were encapsulated in GelMA hydrogels in this experiment. The red fluorescence of MSC-EVs encapsulated in the GelMA hydrogel indicated their successful labeled with CM-DiI (Fig. S2b). After contusive SCI, the dura of the spinal cord was removed carefully by a pair of surgical scissors. Then, the MN patch was placed on top of the spinal cord lesion, and a solution of GelMA mixed with MSC-EVs was mounted on the top of the MN patch, followed by a blue light photocuring process (Fig. 3a). Seven days after surgery, we found that most of the released MSC-EVs were localized and aggregated in the spinal injury site (Fig. 3b, c, b1, b2, c1, c2), indicating the localized delivery capacity of the MN patch. Therefore, we concluded that the MN patch has excellent localized delivery capacity of MSC-EVs in vivo, which facilitates sustained MSC-EV delivery for SCI treatment.

Next, we attempted to assess whether the cells in the MN-MSC patch survived long enough to ameliorate the SCI-induced neuroinflammatory environment and protect the spared tissues/axons from secondary injuries. We implanted the MN patch into the spinal cord lesion of the same SCI model, and the solution of MSCs mixed with GelMA was mounted on the top of the MN patch before the blue light photocuring process was performed (Fig. 3a, S3a–i). As we know, the optimal time for suppressing acute SCI-triggered neuroinflammation and protecting the spared tissue/axons is within 7 days after SCI^23,33^, so we dissected the spinal cord on the 7th day after SCI to assess whether MSCs survived in the MN-MSC patch until this time point. As indicated by the images of spinal sections stained with hematoxylin and eosin (H&E) (Fig. 3d), the MN patch still persisted on the spinal cord surface at this time. More importantly, staining of the human MSC marker GAPDH in the spinal tissues indicated that the MSCs had survived well in the MN-MSC patch on the 7th day after SCI (Fig. 3e, f, red). These results confirmed that the MSCs in the MN-MSC patch can survive at the injury site long enough to cover the optimal therapeutic time window of SCI. Moreover, immunofluorescent staining of a serial spinal section from the spinal border to the center showed that MSCs from the MN-MSC patch did not migrate from the GelMA hydrogel block into the spinal cord (Fig. S4). The results indicated that the fabricated device successfully maintained MSC viability, which may enable sustainable release of MSC-EVs for at least one weeks without MSC invasion into the spinal cord.

### The MN-MSC patch alleviates the inflammatory response in spinal cord lesions after SCI

**Fig. 4:**
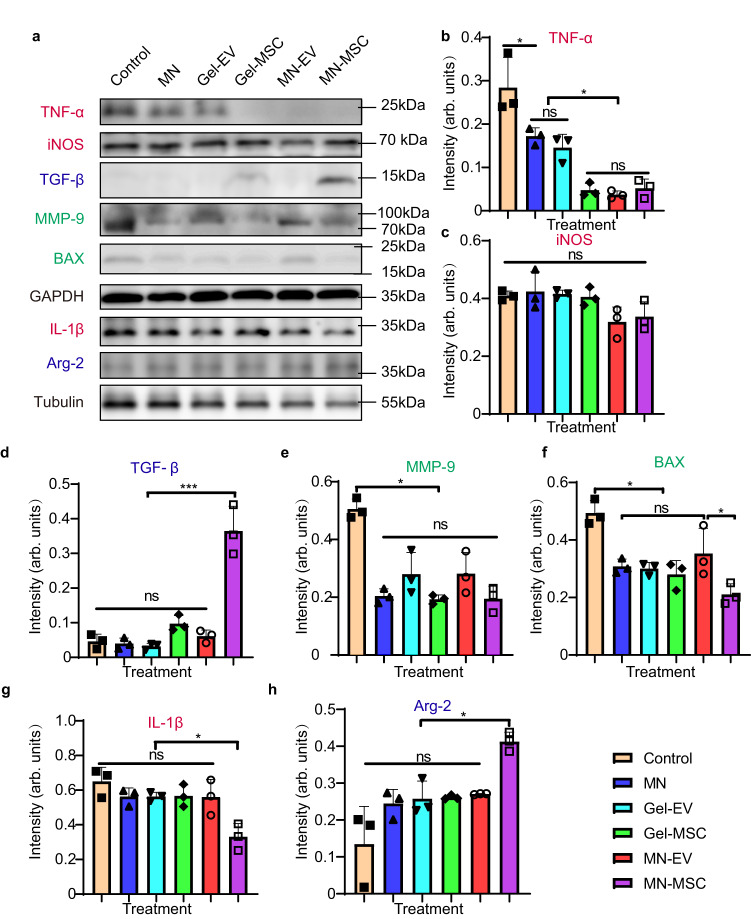
Genetic expression analysis of the injured spinal cord tissues. **a** Representative immunoblots showing expression of TNF-α, iNOS, MMP-9, TGF-β, BAX, IL-1β, and Arg-2 in the injury site 1 week after SCI (red font represents proinflammatory markers, blue font represents anti-inflammatory markers, and green font represents anti-apoptotic/inflammatory markers; GAPDH and Tubulin were used as loading controls). The experiment was repeated 3 times independently with similar results. **b**–**h** Quantitative analyses of the genetic expression levels of TNF-α (**b**), iNOS (**c**), TGF-β (**d**), MMP-9 (**e**), BAX (**f**), IL-1β (**g**), and Arg-2 (**h**) in the injury site. *n* = 3 animals chosen randomly from each group. Data are presented as the mean ± SEM. Statistical analysis was performed using one-way ANOVA followed by Tukey’s multiple comparisons test and two-tailed paired *t* tests were used for comparisons between two groups. ANOVA for TNF-α: Total: *F* = 24.27, *p* < 0.0001, Control vs MN, *p* = 0.0164, Gel-EV vs MN-MSC, *p* = 0.0493. ANOVA for iNOS: Total: *F* = 3.178, *p* = 0.0468. ANOVA for TGF-β: Total: *F* = 43.96, *p* < 0.0001. MN-EV vs. MN-MSC, *p* = <0.0001. ANOVA for MMP-9: Total: *F* = 17.14, *p* < 0.0001. Control vs MN-MSC, *p* < 0.0001. ANOVA for BAX: Total: *F* = 11.18, *p* = 0.0003. Control vs MN-EV, *p* = 0.0391. MN-EV vs MN-MSC, *p* = 0.0373. ANOVA for IL-1β: Total: *F* = 6.960, *p* = 0.0029. MN-EV vs MN-MSC, *p* = 0.0178. ANOVA for Arg-2: Total: *F* = 9.560, *p* = 0.0007. MN-EV vs MN-MSC, *p* = 0.0388. *p < 0.05, **p < 0.01, ***p < 0.001.

To assess whether the MN-MSC patch could ameliorate the SCI-induced neuroinflammatory environment, we constructed animal models without any treatment (control group), with MN patches implantation alone (MN group), with EVs in gel implantation alone (Gel-EVs group), with MSC in gel implantation alone (Gel-MSC group), with MN patches embedded with MSC-EV implantation (MN-EV group) or with MN patches seeded with MSC implantation (MN-MSC group) for comparison. 7 day after transplantation, we performed Western blotting analysis to detect the protein expression levels of the spinal tissues with spinal cord lesions in rats that underwent different treatments. In the spinal cord tissues of rats treated with the MN-MSC patch, we observed a significant decrease in the expression of proinflammatory markers IL-1β and TNF-α (Fig. 4a, b, g) and an increase in expression of the anti-inflammatory markers TGF-β and Arg-2 (Fig. 4a, d, h). Moreover, the expression of the proapoptotic marker BAX and the inflammatory marker MMP-9 was significantly reduced (Fig. 4a, e, f). Our experiments demonstrated that the MN alone exhibited some effect in reducing excessive TNF-α and MMP-9 expression (Fig. 4a, b, e), primarily controlled by M1 macrophages, compared to the control group (Fig. 4). All groups with the MN patch treatments exhibited decreased TNF-α and BAX expression, indicating suppressed inflammation. However, only the MN-MSC treatment yielded a statistically significant increase in the anti-inflammatory cytokine TGF-β as well as increased arginase-2 and decreased IL-1β expression. These findings suggest the beneficial effects observed could be partly attributable to a physiological reaction to the MN patch itself.

To further analyze the tissue-protective efficacy, sagittal sections of injured spinal cords at 8 weeks post-SCI were analyzed with immunofluorescence staining. Immunostaining for Iba1 (macrophages/microglia) showed significantly fewer activated immune cells in rats receiving the MN- MSC patch treatment compared with control or MN-EV groups (Fig. S5a, e). In contrast, more neurons (NeuN) (Fig. S5b, f), blood vessels (CD31) (Fig. S5c, g), myelin (MBP) (Fig. S5d, h) and astrocytes (GFAP) (Fig. S5a–d, i) were observed in spinal tissues of the rats in the MN-MSC group. Thus, evidence of cell preservation was only observed in the MN-MSC treatment condition.

### The MN-MSC Patch Protects Spared Tissues/Axons from Secondary Injury

**Fig. 5:**
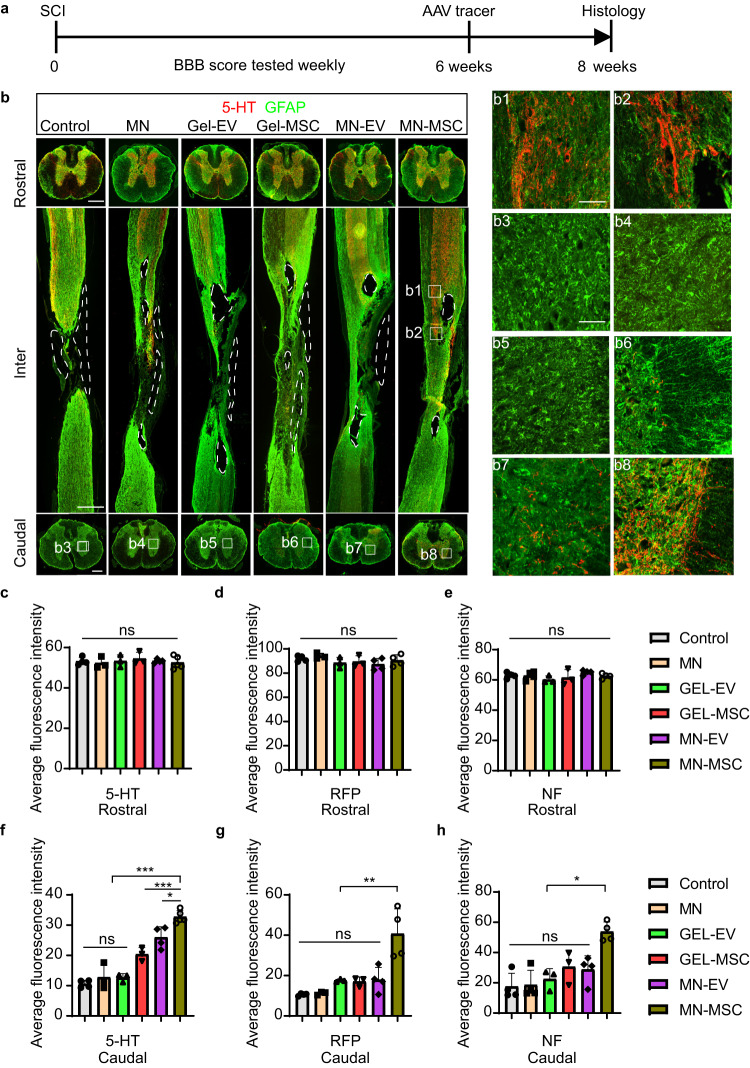
Protection of spared axons with MN-MSC patch treatment after SCI. **a** Schematic diagram of the experimental design. The MN or MN-MSC patch was implanted 3 h after SCI, the AAV tracer for axons was injected at 6 weeks after injury, and the histological study was performed at 8 weeks after injury. **b** Representative images of spinal sections stained with GFAP (green) and 5-HT (red) of rats in the six groups at 8 weeks after SCI. Solid lines indicate the boundary of the cavities. Scale bars for rostral and caudal indicate 500 µm and inter indicate 1 mm. b1–b8 show the details of the black boundary area, and the scale bars are 100 µm. Representative images of propriospinal axons (RFP labeled, Fig. S6) and NF axons (Fig. S7) in SCI rats with different treatments are shown in supplementary figures. **c**–**h** Quantification of the average fluorescence intensity of 5-HT (**c**), RFP (**d**), and NF (**e**) immunoreactivity on the rostral and caudal sides of the six groups with 5-HT (**f**), RFP (**g**), and NF (**h**) staining. Data are shown as the mean ± SEM. Statistical analysis was performed using one-way ANOVA followed by Tukey’s multiple comparisons test and two-tailed paired *t* tests were used for comparisons between two groups. Control: *n*_5-HT_ = 4, *n*_RFP_ = 4, *n*_NF_ = 4. MN: *n*_5-HT_ = 3, *n*_RFP_ = 3, *n*_NF_ = 4. Gel-EV: *n*_5-HT_ = 3, *n*_RFP_ = 3, *n*_NF_ = 3. Gel-MN: *n*_5-HT_ = 3, *n*_RFP_ = 3, *n*_NF_ = 3. MN-EV: *n*_5-HT_ = 4, *n*_RFP_ = 4, *n*_NF_ = 4. MN-MSC: *n*_5-HT_ = 5, *n*_RFP_ = 4, *n*_NF_ = 4. These animals chosen randomly from each group. ANOVA for 5-HT of rostral: Total: *F* = 0.2836, *p* = 0.9152. ANOVA for 5-HT of caudal: Total: *F* = 47.76, *p* < 0.0001, Control vs MN-MSC, *p* < 0.0001, GEL-MSC vs MN-MSC, *p* < 0.0001, MN-EV vs MN-MSC, *p* = 0.0134. ANOVA for RFP of rostral: Total: *F* = 1.243, *p* = 0.3380. ANOVA for RFP of caudal: Total: *F* = 12.13, *p* < 0.0001, MN-EV vs MN-MSC, *p* = 0.0013. ANOVA for NF of rostral: Total: *F* = 1.167, *p* = 0.3675. ANOVA for NF of caudal: Total: *F* = 9.322, *p* = 0.0003, MN-EV vs MN-MSC, *p* = 0.01. *p < 0.05, **p < 0.01, ***p < 0.001.

After verifying that MN-MSC treatment could reduce SCI-triggered inflammation, we sought to assess whether this treatment could protect spared tissues/axons from secondary injury after SCI. To determine whether more descending axons survived after being treated with an MN-MSC patch, we injected AAV2/9-hSyn-mCherry into the T7-T8 region of the 6 groups of rats 2 weeks before sacrifice to trace the descending propriospinal axons (Fig. 5a). After staining the spinal sections with a red fluorescent protein (RFP) antibody (Fig. S6), we found no significant difference between numbers of RFP^+^ axons above the lesion site in the different treatment groups (Fig. 5d). However, rats in the MN-MSC group had higher numbers of RFP^+^ propriospinal axons below the injury level compared to other groups (Fig. 5g).

Then, we stained the serotonergic axons and axonal fibers with anti-5-hydroxy tryptamine (5-HT) and anti-neurofilament (NF) in the spinal sections, respectively. We found that the trends of 5-HT and NF staining results were similar to that of RFP staining (Fig. 5, S7). No significant difference between NF and 5-HT was found above the injury site in the six groups (Fig. 5c, 3e, S7). In contrast, the rats in the MN-MSC group had more serotonergic axons (stained by 5-HT) and axonal fibers (stained by NF) below the injury site than the rats in the other groups (Fig. 5f, 3h, S7), indicating that more propriospinal axons survived after SCI with MN-MSC patch treatment. In contrast to the other groups, we found more axons that extended into the spinal cord lesion site in the examined subjects with MN-MSC treatment (Fig. 5b and S7), suggesting that the microenvironment with the sustained EVs delivery from MN-MSC patch treatment was more conducive to axon regrowth. In addition, we did not observe any significant difference between the control and MN groups in terms of protecting spared axons from secondary injury (Fig. 5, S6, S7). These results suggest that the microneedle arrays themselves did not have any effect on axonal protection. Interestingly, we did observe more 5-HT axons present beneath the injury in the rats with Gel-MSC and Gel-EVs treatment compared with these with control, MN, Gel-EV treatments (Fig. 5b, f), suggesting these treatments exhibit some, albeit limited, neuroprotective effects. Remarkably, we observe a significantly higher number of axons situated beneath the injury site in the MN-MSC group compared to the other groups (Fig. 5, S6, S7). These findings highlight the essential contribution of the microneedle patch to our treatment.

Recent studies on MSCs have reported that injecting MSCs directly into the vein (IV) or the damaged area (in situ) of the spinal cord can lead to some recovery in patients or animal models with SCI^11,12,15^. To accurately compare the effectiveness of IV and local transplanted treatments, we conducted a study using both IV injection and in situ injection of MSCs alone as two additional controls. This allowed us to evaluate the ability of MN-MSCs to improve SCI under the same model and severity of injury (Fig. S8a). We found that the treatment with MSCs alone locally or through IV injection did not demonstrate any significant differences from the control group (Fig. S8b–d, S9).

### The remnants of spared axons mediate hindlimb locomotor functional recovery

**Fig. 6:**
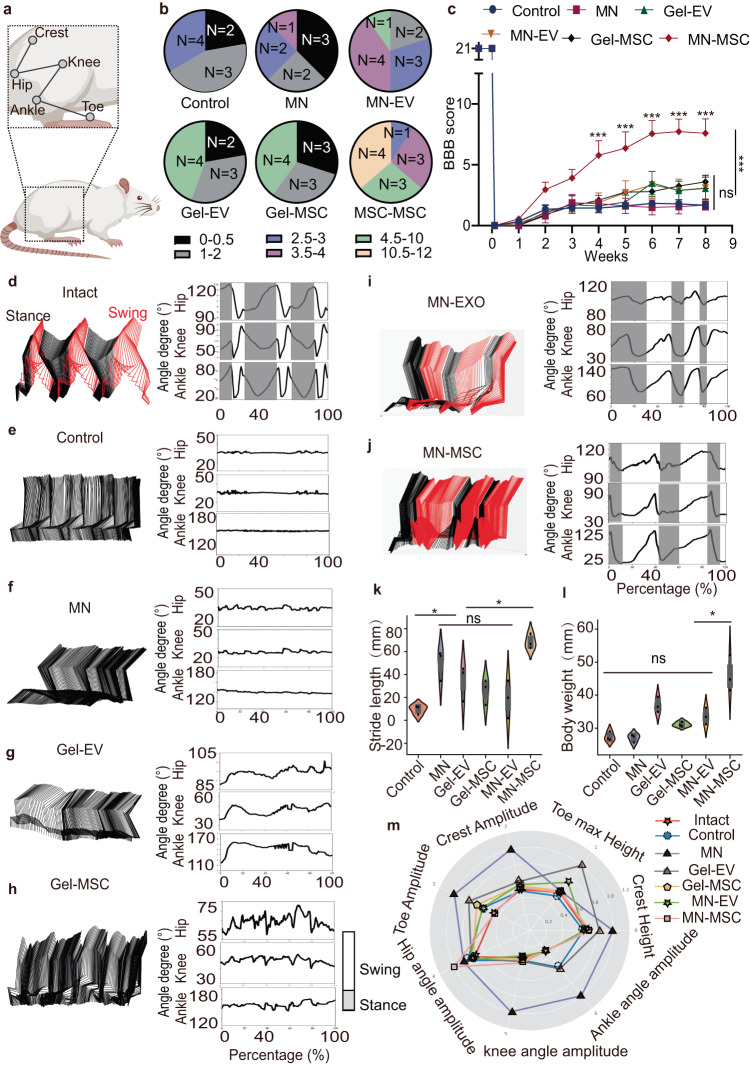
The hindlimb locomotor functional recovery of rats that underwent MN-MSC patch treatment after SCI. **a** Schematic illustration of hindlimb locomotor joint. **b** Distribution of BBB scores of rats in six treatment groups at 8 weeks after SCI. **c** Comparison of weekly BBB scores among rats in the six treatment groups at 8 weeks after SCI. Data are shown as the mean + SEM. Two-way ANOVA with Tukey’s post-hoc test was used for comparisons among multiple groups, and two-tailed paired *t* tests were used for comparisons between two groups. Control: *n* = 9, MN: *n* = 8, Gel-EV: *n* = 9, Gel-MSC: *n* = 10, MN-EV: *n* = 10, MN-MSC: *n* = 11 independent animals. Total: *F* = 2.928, *p* < 0.0001. 4 weeks: MN-EV vs MN-MSC, *p* < 0.0001. 5 weeks: MN-EV vs MN-MSC, *p* < 0.0001. 4 weeks: MN-EV vs MN-MSC, *p* < 0.0001. 5 weeks: MN-EV vs MN-MSC, *p* = 0.0001. 6 weeks: MN-EV vs MN-MSC, *p* < 0.0001. 7 weeks: MN-EV vs MN-MSC, *p* < 0.0001. 8 weeks: MN-EV vs MN-MSC, *p* < 0.0001. *p < 0.05, **p < 0.01, ***p < 0.001. **d**–**j** Color-coded stick views and angle degree curves from intact (**d**), control (**e**), MN (**f**), Gel-EV (**g**), Gel-MSC (**h**), MN-EV (**i**), and MN-MSC (**j**) groups. **k**, **l** Quantitative analysis of body strike length (**k**) and weight support (**l**). One-way ANOVA followed by Tukey’s post-hoc test was used for comparisons among multiple groups and two-tailed paired *t* tests were used for comparisons between two groups. *n* = 3 independent animals. ANOVA for body strike length, Total: *F* = 9.771, *p* = 0.0007. Control vs MN, *p* = 0.0142, MN-EV vs MN-MSC, *p* = 0.0028. ANOVA for weight support, Total: *F* = 20.31, *p* < 0.0001, MN-EV vs MN-MSC, *p* = 0.0013. *p < 0.05, **p < 0.01, ***p < 0.001. The violin plot center indicates the median in all planes. Violin range covers 97.5th and 2.5th percentiles; extending whiskers show data distribution and probability density. Violin areas remain constant. Boxplot centerlines signify medians; boxes show first and third quartiles (Q1, Q3); whiskers extend from Q1 − 1.5xIQR to Q3 + 1.5xIQR; outliers lie outside whiskers. **m** Radar graph quantification of seven behavioral features of rats in the six treatment groups. For more details, see Fig. S10.

After verifying that more spared axons could be rescued by MN-MSC patch treatment, we sought to determine whether these axons could mediate hindlimb locomotor functions. To this end, we analyzed the hindlimb performance of the rats with the Basso, Beattie, and Bresnahan (BBB) scale in a double-blind manner^34^. We found that the rats in the other groups exhibited paralysis with occasional ankle movement during the observation period, with an average BBB score of less than 4 (Fig. 6c). Even though the rats in the MN-MSC group predominantly demonstrated paralyzed hindlimbs during the first 3 weeks and showed no significant difference from the other groups (Fig. 6c), they showed vigorous knee and ankle movement at 4 weeks. The BBB scores of these rats were significantly higher than those of rats in the other groups at 4 weeks after injury. More strikingly, at the 8th week after SCI, the rats treated with the MN-MSC patch showed improved hindlimb motor function, with higher body weight support and stride length than the rats in the other groups (Fig. 6k–i). Remarkably, we found that 4 of 11 rats in the MN-MSC patch-treated group showed sustained weight support with hindpaw plantar stepping, and the BBB score reached more than 10, which was not observed in the rats in the other groups (Fig. 6b, Supplementary movie 1). Moreover, through subsequent analysis of the 7 kinds of hindlimb kinematics^35^ of rats in the control, MN, Gel-EV, Gel-MSC, MN-EV, MN-MSC and intact groups, we found that the MN-MSC patch treatment could increase the maximal height of the iliac crest and toe (Fig. S10a, b); the height amplitude of the iliac crest and toe (Fig. S10c, d);. Interestingly, we observed an increased stride length in rats treated with MN, Gel-EV, Gel-MSC, and Gel-EV compared to the control group, suggesting that the limited neuroprotective effects from MN may also contribute to their modest recovery (Fig. 6k). Meanwhile, in the Gel-EV, Gel-MSC, and Gel-EV groups, the angle oscillation of the hip, knee, and ankle were larger than those in the MN group and comparable to the MN-MSC group (Fig. S10e–g). However, the hindlimb locomotor performance of the rats treated with the MN-MSC patch was significantly better than that of rats in the other groups but not as good as that of the rats in the intact group, suggesting that the hindlimb functional recovery with this treatment relied on the remnants of spinal connections across the lesion.

To take both features of data distribution and statistical meaning into account in each kinematics indicator, we drew a radar plot (Fig. 6m). While all indicators in each group were divided by the counterpart in the intact group, it was obvious that the amplitudes of the crest and toe of the rats in the MN-MSC group were fairly high compared with those of rats in the other treatment groups (Fig. 6e–j). However, the maximum heights of the toe and crest of the rats treated with MN-MSC were remarkably higher than those of rats in the other groups (Fig. 6m), possibly because the crest and toe determine the gait and stride length while walking. Upon comprehensively considering the values and distributions for each group, we found that angle amplitude changes at the ankles, knees and toes remained minimal in rats of the MN-MSC group. This is consistent with results of the hindlimb kinematic analysis (Fig. S10).

**Fig. 7:**
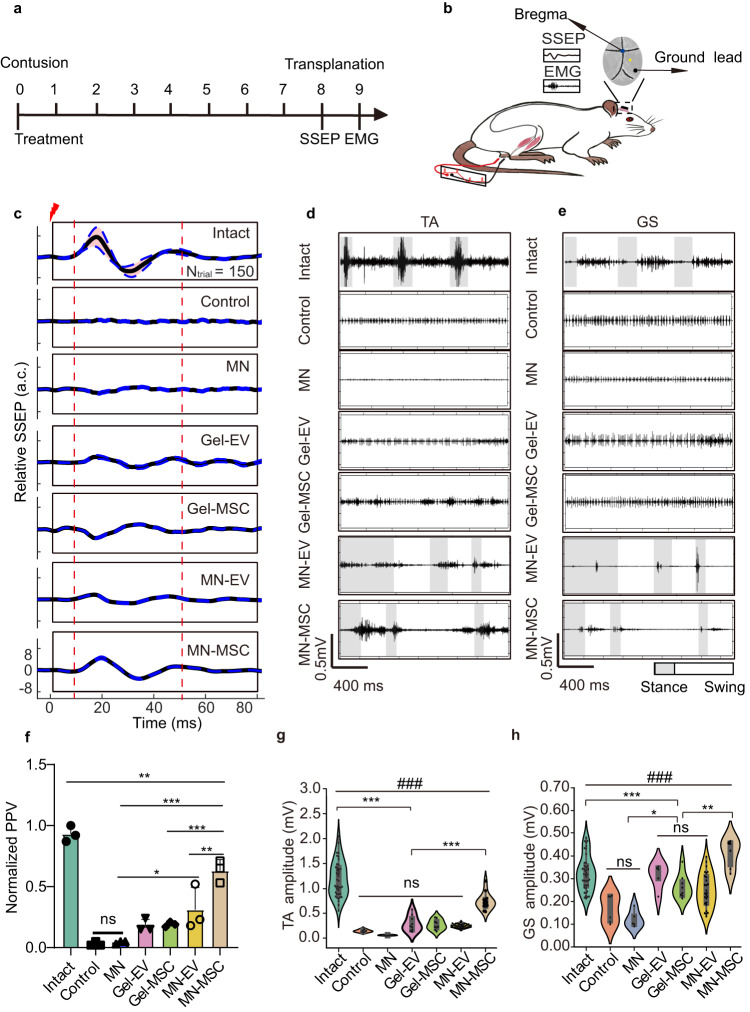
Electrophysiological recording experiments revealed the superior muscle control of rats treated with the MN-MSC patch. **a** Schematic diagram of the experimental design. **b** Schematic illustration of SEEP and EMG experiments. **c** Representative relative SSEPs of each group (*N* trial = 150) are illustrated as the mean (solid line) and 95% confidence interval (shadow enveloped by a dashed line), and the time of electric stimulation was set to zero ms. **d**, **e** The representative TA(d) and GS(e) muscle EMG for rats of different groups. **f** Peak-to-peak potential was analyzed with one-way ANOVA followed by Tukey’s post-hoc test. Two-tailed paired *t* tests were used for comparisons between two groups. Data are shown as the mean ± SEM. *n* = 3 animals chosen randomly from each group. Total: *F* = 46.59, *p* < 0.0001, MN vs MN-EV, *p* = 0.0221, Intact vs MN-MSC, *p* = 0.0092, MN vs MN-MSC, *p* < 0.0001, Gel-MSC vs MN-MSC, *p* = 0.0003, MN-EV vs MN-MSC, *p* = 0.0054. *p < 0.05, **p < 0.01, ***p < 0.001. **g**, **h** Quantitative analysis of signal amplitudes from TA(g) and GS(h) muscles for rats in different groups. One-way ANOVA with Tukey’s post-hoc test for comparisons among multiple groups (*) and two-tailed paired *t* tests were used for comparisons within groups (#) for the data shown in the violin plot. The violin plot center indicates the median in all planes. Violin range covers 97.5th and 2.5th percentiles; extending whiskers show data distribution and probability density. Violin areas remain constant. Boxplot centerlines signify medians; boxes show first and third quartiles (Q1, Q3); whiskers extend from Q1 − 1.5xIQR to Q3 + 1.5xIQR; outliers lie outside whiskers. Intact: *n*_TA_ = 41, *n*_GS_ = 41, control: *n*_TA_ = 6, *n*_GS_ = 6, MN: *n*_TA_ = 6, *n*_GS_ = 5, Gel-EV: *n*_TA_ = 9, *n*_GS_ = 5, Gel-MSC: *n*_TA_ = 9, *n*_GS_ = 8, MN-EV: *n*_TA_ = 35, *n*_GS_ = 27, and MN-MSC: *n*_TA_ = 13, *n*_GS_ = 6 trials for each group. ANOVA for TA: Total: *F* = 78.441794, *p* = 8.507914 × 10^−38^, Intact vs MN-EV, *p* < 0.0001. MN-EV vs MN-MSC, *p* = 9.853309 × 10^−9^. ANOVA for GS: Total: *F* = 13.718067, *p* = 4.86509 × 10^−11^. Intact vs MN-EV, *p* = 8.533763 × 10^−4^. MN vs MN-EV, *p* = 4.474681 × 10^−3^. MN-EV vs MN-MSC, *p* = 4.238509 × 10^−5^. ***p (or ###p) <0.001, **p (or ##p) <0.01, *p (or #p) < 0.05.

To further determine the mechanism of action of the MN-MSC patch treatment, we performed a somatosensory evoked potential (SSEP) monitoring study of the rats during anesthesia (Fig. 7b, c, f). Compared with the BBB score, SSEP is more direct for measuring the functionality of neuropathways from cortical projections^33^. As expected, we found that the SSEPs showed a similar trend to the BBB scores at the 8th-week post-injury (Fig. 7c, f), which confirms the axon protection capability of MN-MSC patch treatment. Then, we recorded electromyography (EMG) data to assess the rats’ hindlimb muscle motion when they were walking. We found that the ankle flexor signals of tibialis anterior (TA) muscles and extensor gastrocnemius soleus (GS) muscles were rarely active in rats in the control group, and the muscle signals of other treatment groups could only be occasionally observed. In contrast, alternating activation of the TA and GS for the MN-MSC patch-treated rats could be consistently observed during different steps (Fig. 7d, e, g, h). To further analyze the EMG data, astronomical analysis (Poincaré statistical analysis) was used to analyze the rhythm of the TA and GS muscles^36^. We found that the rhythms of TA and GS muscles of the rats in the MN-MSC group were more regular than those of rats in the other groups with a wider distribution range of scatter points and more concentrated at the central points (zero) position, which were more similar to the one of the intact group (Fig. S11, S12), suggesting that those rats gain much better muscle control through MN-MSC patch treatment. From this, we explored why the gaits of rats treated with MN-MSC patches were closer to normal than the gaits of other rats. Together, these results suggest that improved locomotor function in the MN-MSC patch treatment condition is mediated by axon rescue.

## Discussion

In this study, we constructed an advanced MN-MSC patch that can provide sustained delivery of MSC-EVs from the MSC-embedded patch to the spinal tissues in the injury site and avoid the risk of direct stem cells invading the spinal cord. The MN component of the patch was made of porous GelMA hydrogel with good biocompatibility, and the patch can remain on top of the spinal cord lesion for a long time, thus encompassing the optimal therapeutic window and achieving the maximum therapeutic efficacy of MSC-EV treatment.

The study proposed an advanced MN-MSC patch for SCI treatment, providing a platform for the long-term survival of MSCs and enabling efficient and precise sustained delivery of MSC-EVs to SCI lesions for an extended period, without direct invasion of MSCs into the spinal cord microenvironment, thereby reducing the potential for additional damage from in situ transplantation. Traditional MSC-EVs therapies were always hindered by the brief retention time of MSC-EVs in living subjects^9,22^. While, our data suggest that treatment with sustained MSC-EVs by MN-MSC patch after SCI could reduce secondary injury effectively^10,15,33^. Previous studies have shown that MSCs undergo apoptosis quickly when transplanted into the injury site in the presence of cytotoxic cells^37–39^. However, in our MN patch, MSCs are kept in a relatively moderated microenvironment after transplantation, and their ability to produce MSC-EVs might be more stable, allowing them to exert a neuroprotective effect. MN-MSC patches seeded with MSCs provide an approach for achieving greater sustained delivery of MSC-EVs for at least seven days after SCI than direct injection of MSCs or MSC-EVs. Furthermore, in this SCI model, MN-MSC seeded patches effectively down regulated neuroinflammation after SCI, resulting in greater tissue sparing.

In this study, we double-blindly evaluated functional recovery in a model of severe contusive SCI. Our findings showed that MN-MSC patch treatment could promote functional recovery by protecting the spared axons from secondary injury. Consistent with the MSC secretome’s function of immune modulation, apoptosis inhibition^17,40^ and enhanced angiogenesis^20,41,42^, greater vascularization (CD31) formed in the spinal cord lesion of SCI rats with our treatment. Such vessels can supply sufficient nutrients and oxygen to remodel the SCI microenvironment and then protect spared axons^15,33,43^. Furthermore, the spinal cord of rats treated with the MN-MSC patch showed less inflammation (Iba1, TNF-α, IL-1β, MMP-9) and more anti-inflammatory factors (TGF-β, Arg-2) with more neurons (NeuN), less apoptosis and death of spinal cord cells (BAX), and more myelin sheath (MBP), which benefit the plasticity of spinal circuitry in terms of function. While some groups with the MN patch also benefitted by modulating the excessive immune response, this effect could be attributable to the inherent properties of GelMA in regulating the neuroinflammatory microenvironment. No significant differences were observed between the Gel-EVs and MN groups, potentially due to the limited therapeutic effects of Gel-EVs (Fig. 4a, b, e). Furthermore, the Gel-MSC and MN-EV groups demonstrated enhanced immunosuppression by decreasing TNF-α levels compared to the MN groups (Fig. 4a, e). These results suggest the MN-MSC treatment shifts the pro-inflammatory and anti-inflammatory balance from an M1 macrophage-dominant state (as in control groups) to an M2 macrophage-dominant state^22^, possibly due to sustained MSC-EVs delivery by the MN-MSC patch. These findings highlight the need for both microneedles to enable effective delivery and MSCs to achieve sustained MSC-EVs delivery, thereby accomplishing reliable immunomodulation.

Additionally, by axon tracing and labeling, we showed that more axons survived secondary injury by MN-MSC patch treatment. In comparison with the rats in the control or other treatment groups, the rats with MN-MSC patch treatment exhibited remarkable hindlimb locomotion functional recovery, which is consistent with their better muscle control ability, as indicated by the PPV and signal of TA and GS muscle EMG recording studies. However, a variety of functional recovery levels was observed in the rats treated by MN-MSC patches, possibly because the rescued axons that are closely relevant to hindlimb locomotion were randomly presented in the treated rats. The therapeutic effects observed in this study may be explained by the effective vascularization (Fig. S5) and inflammatory mitigation (Fig. 4) elicited by the MSC-EVs.

Although MSCs release many soluble proteins in addition to EVs, preliminary studies with a small number or animals found that the addition of GW4869^44^ (which block exosome release) to the MN-MSC patch blocked the histological and functional improvements observed with MN-MSC patches without GW4869 (Fig. S14). This implies that release of EVs from the implanted MSCs may be necessary for the therapeutic effects. However, we cannot rule out the possibility that tropic factors or other soluble molecules might contribute to the effects. Additionally, we cannot exclude the possibility that these factors coexist and synergize with the generation of MSC-EVs^45–47^. The interplay between MSC-EVs and other factors, such as trophic factors, remains one of the main challenges in the MSC therapy field and awaits further investigation^10,15^. Our findings indicate that the sustained release of EVs is crucial, but we cannot exclude the possibility that other factors, such as releasing a greater total number of EVs or delayed release, may also contribute to the observed therapeutic effects of the MN-MSC patch. Other strategies are needed to better preserve transplanted MSCs’ stemness, thereby improving treatment efficacy. These remain the limitations of this study.

While we have demonstrated the proof-of-concept for the MN-MSC patch in SCI treatment, the optimal time window for MN-MSC patch implantation remains to be determined. In an additional experiment, MN-MSC patch implantation was performed on the 3^rd^, 7^th^, and 14^th^ days following contusive SCI. Associated data shows that the application of the MN MSC patch at 3 hr, 3 days, or 7 days post-SCI resulted in axon sparing below the lesion and improved locomotor recovery, but that treatment at 14 days did not do either (Fig. S13). The proposed MN-MSC transplantation treatment is likely effective within at least the first week after SCI, in line with previous studies indicating that the optimal transplantation time window for neuroprotective treatments falls within the first week post-SCI^10,16,34^. Moreover, in contrast to the MN-MSC treatment conditions, in our hands, IV infusion or direct transplantation of 1.0 × 10^6^ MSCs one week post-injury failed to produce statistically significant improvements in locomotor recovery or axon sparing (Fig. S8), arguing that the MN scaffold may provide for superior survival and engraftment of MSCs. However, optimizing treatment timing requires further investigation.

Since the MN-MSC patch was fabricated from GelMA hydrogel, we envisioned that the MN-MSC patch would degrade in the long term^29,48^. However, whether the materials of the MN-MSC patch could completely degrade should be investigated in future studies. In addition, the relationship between the number of EVs released from the MN-MSC patch and the best treatment efficacy has not been tested and therefore needs to be investigated before clinical study. While our study demonstrated that MN-MSC transplantation was effective within the first week following SCI, further optimization of the transplantation time window may be necessary for pre-clinical exploration. To promote diffusion and improve delivery efficiency, in our next step, we plan to construct a precise porous MN structure at the micro/nano scale with two-photon 3D printing technology, which might have higher precision in controlling the porous size of the MNs^49^. Moreover, the MN-MSC patch might be too soft to be used as an artificial dura for clinical SCI treatment, and a tenacious MN-MSC patch with the addition of a scaffold structure between the MN array and the MSC-embedded patch is being developed by our group.

## Methods

### Materials

Chicken anti-GFAP [abcam (ab134436, 1:500)], GAPDH (Human Specific) Rabbit mAb [Abclonal (AC036), 1:500], rabbit anti-neurofilament (NF) heavy polypeptide [abcam (ab8135), 1:500], goat anti 5-HT antibody [abcam (ab66047), 1:500], rabbit anti-NeuN [abcam (ab177487), 1:1000], Rabbit polyclonal to RFP[abcam (ab62341), 1:500] rabbit anti-MBP [abcam (ab218011), 1:500], goat anti-IBA1 [abcam(ab5076), 1:500] rabbit anti-CD31 [R&Drd system (AF3628), 1:500], GAPDH Rabbit Monoclonal Antibody [Beyotime (AF1186,1:1000)] rabbit anti-TGF- β[Abclonal(A2124), 1:1000], rabbit anti-Bax [Abclonal (A0207),1:1000], rabbit anti-MMP9 [Abclonal (A0289), 1:1000], rabbit anti-Arginase 2 (ARG2) [Abclonal (A19233), 1:1000], Donkey anti-Chicken IgY H&L (FITC) [Abcam (ab63507), 1:500], Donkey anti-Rabbit IgG H&L (Alexa Fluor® 555) [Abcam (ab150062), 1:500], Rabbit anti-Goat IgG H&L (Alexa Fluor® 555) [Abcam (ab150142), 1:500], Donkey anti-Rabbit secondary antibodies (HRP) [beyotime (A0208), 1:1000], Rabbit anti-iNOS [proteintech (80517-1-RR), 1:2000] Rabbit anti-TNF-α [proteintech (17590-1-AP), 1:1000], Rabbit anti-IL-1β [Abclonal (A20527), 1:1000] Rabbit α-Tubulin [beyotime (AG0126), 1:1000] were used. PE Mouse anti-Human CD105 [BD Pharmingen™, 560839,1:20], APC Mouse Anti-Human CD90 [BD Pharmingen™, 561971, 1:20], PE Mouse Anti-Human CD73 [BD Pharmingen™, 550257, 1:20], FITC Mouse Anti-Human CD45 [BD Pharmingen™, 561865, 1:20], FITC Mouse Anti-Human CD34 [BD Pharmingen™, 560942, 1:20], APC Mouse Anti-Human CD19 [BD Pharmingen™, 561742, 1:20], APC Mouse Anti-Human CD14 [BD Pharmingen™,555399, 1:20], PE Mouse Anti-Human CD11b [BD Pharmingen™, 555388, 1:20], PE Mouse Anti-Human CD79a [BD Pharmingen™, 561942, 1:20], PE Mouse Anti-Human HLA-DR [BD Pharmingen™, 555812, 1:20] were used. The adenovirus-associated virus AAV2/9-hSyn-mCherry was generated by the viral core of Zhejiang University, and its titer was adjusted to 1 × 10^13^ copies per mL for injection. DAPI-containing antifade mounting medium was purchased from Southern Biotech (0100-20, USA). Triton X-100 (P1080) was purchased from Solarbio (China). Lithiumphenyl-2, 4, 6-trimethylbenzoylphosphinate (LAP), polydimethylsiloxane molds (EFL-MMN-600), traditional GelMA (EFL-GM-PR-001) and porous GelMA (EFL-GM-PR-001) were purchased from EFL Inc. (Suzhou, China). GW4869 were obtained from the Sigma-Aldrich. Serum-free medium for MSCs (NC0103 + NC0103. S, Yocon, Beijing, China). Serum-free medium for MSCs and stem cells moderate digestive enzymes were bought from Yocon. CCK-8 assay was bought from Dojindo. Live/dead viability/cytotoxicity kit was bought from Invitrogen. CM-DiI was bought from Yeasen.

### Animals

Female Sprague‒Dawley rats (220-250 g) were purchased from the Zhejiang Academy of Medical Sciences. All animal experiments were approved by the Institutional Animal Care and used following the provisions of Zhejiang University Animal Experimentation Committee and Independent Ethics Committee (ZJU202010110). The rats were housed under controlled environmental conditions and were completely in compliance with the National Institutes of Health Guide for the Care and Use of Laboratory Animals. The implantation of electrodes was performed under anesthesia with dexmedetomidine hydrochloride (0.25 mg/kg i.p.; MCE, US) and 0.5% isoflurane. All the other surgeries were performed under anesthesia with 1% (w/v) pentobarbital sodium (5 mL/kg, injected intraperitoneally).

### Preparation of MSC

Human embryonic stem cell (ESC)-derived MSCs were generated by Ysbiotech, Hangzhou, China (YS™ hESC-MSC) and cultured at 37 °C in a humidified atmosphere of 5% CO_2_ in serum-free medium for MSCs (NC0103 + NC0103. S, Yocon, Beijing, China) as previously reported^50^, with some modification. The H9 human embryonic stem cell line (WA09), originally generated by the National Stem Cell Bank c/o WiCell Research Institute (USA), was obtained from the stem cell bank at the Institute of Biochemistry and Cell Biology, CAS. ESC-MSCs were differentiated from ESC as following the method. Briefly, the H9-ESC colonies were dissociated into small clumps using TrypLE Express and cultured in ultralow-attachment plates with E8 medium (Gibco, Grand Island, NY, USA). After one week, we harvested the embryoid bodies and cultured them in MSC induction medium (high-glucose Dulbecco’s modified Eagle’s medium, 10% fetal bovine serum, and 1 mM L-glutamine). After 15 days, the outgrowths of embryoid bodies were sub-cultured using TrypLE Express, yielding MSCs designated as passage 0. The MSCs were subsequently cultured in serum-free medium. When the proliferating colonies had reached near confluence, the hESC-MSCs were passaged using stem cells moderate digestive enzymes (NC1004, Yocon, Beijing, China). After 4 passages, MSCs were used for transplantation or other investigations.

To confirm the MSC phenotype of the obtained cells, we analyzed their CD immuno-profile using flow cytometry (the flow cytometric gating strategy see Fig. S15–17). Specifically, we used flow cytometry to assess the expression of CD73, CD90, CD105, CD45, CD34, CD19, CD14, CD11b, CD79a, and HLA-DR to make sure their MSC phenotypes (results see Fig. S1, CD73^+^, CD90^+^, CD105^+^, CD45^-^, CD34^-^, CD19^-^, CD14^-^, CD11b^-^, CD79a^-^, and HLA-DR^-^)^38,50^.

### Preparation of Extracellular Vesicles

We employed a modified differential centrifugation method to isolate extracellular vesicles from MSCs^51^. Briefly, when the MSCs reached 80-90% confluence, we washed them with PBS and added complete medium containing exosome-free medium for 48 h. The supernatant was collected and subjected to centrifugation at 350 *g* for 15 min and 2300 *g* for 20 min at 4 °C to remove cells and cellular fragments. After filtration through a 0.22 μm filter, the medium was ultra-centrifuged at 120,000 *g* for an additional 2 h at 4 °C to collect MSC extracellular vesicles. The vesicles were then resuspended in 100 μL of sterile PBS. We analyzed the size and morphology of the extracellular vesicles using transmission electron microscopy (TEM) with a JEM-1400flash microscope (JEOL, Japan). We quantified the extracellular vesicles using nanoparticle tracking analysis (Marvin, UK, Nanosight NS500). Specifically, we first diluted the obtained solution suspended in PBS (approximately 2 μg/μL) by 100-fold and then analyzed it based on light scattering using an optical microscope aligned perpendicularly to the beam axis. Finally, we quantified the vesicles using NTA software.

### Fabrication and characterization of MN arrays

The MN arrays were fabricated with polydimethylsiloxane molds. GelMA (EFL-GM-60) and porous GelMA (EFL-GM-PR-001) were used to create traditional MN arrays and porous MN arrays, respectively. The traditional GelMA (EFL-GM-60) comprises Gelatin methacryloyl (GelMA, Mw ~150 kDa, degree of substitution: 60%) and lithiumphenyl-2, 4, 6-trimethylbenzoylphosphinate (LAP) in a mass ratio of 100/5. The porous GelMA (EFL-GM-PR-001) consists of GelMA (Mw 150 kDa, degree of substitution: 60%), polyethylene oxide (Mw = 300 kDa), and photo-initiator LAP in a mass ratio of 100/15/5. 5% (w/v) solution of EFL-GM-60/ EFL-GM-PR-001 were selected to fabricate the Microneedles array according to previous report^29,30^. To create the MN arrays, we first prepared a 5% (w/v) solution of either traditional GelMA or porous GelMA at 37 °C and dispensed it into molds. Next, the bubbles of the casting solution were removed by vacuum pumping, and the molds were placed in a 37 °C environment for 10 h to dry. Then, the casting solution was solidified with blue light curing (405 nm, 30 mW/cm^2^) for 30 s. After that, the MN was separated from the molds with tweezers cautiously. The MNs had diameters of 250 μm, which tapers for a height of 600 μm. The MN arrays were arranged 550 µm, tip to tip, in a 4 mm × 4 mm area. The morphology of the MNs was assessed with a SEM (Nova Nano 450, Therom FEI, USA).

The compressive mechanical properties of the dry traditional GelMA and porous GelMA gel scaffolds were tested using a texture analyzer (Stable microsystem, TA-XTPLUS, UK). Prior to testing, the samples were soaked in PBS for 4 h. The preload was set to 0.1 N and the maximum compression strain was set to 80%. The stress-strain curve was obtained, and the compression modulus of the sample was calculated based on the slope of the curves in the range of 0-30% deformation. Three samples were tested in each group. The mechanical properties of the MN were tested using a universal testing machine (CMT5205, MTS, China). The MN patches were placed on the test bench with the tip of the needles facing upwards. The compress parameter was set to 0.005 mm/s, and the measured force and displacement were recorded. Three samples were tested in each group.

### Fabrication of the MN-MSC, MN-EV patch, Gel-EV and Gel-MSC

First, the MSCs were digested by stem cell digestive enzymes and evenly mixed into a 5% (w/v) porous GelMA solution with final concentration of about 2.5 × 10^7^ cells/mL at 37 °C. Next, the 40 μL solution with MSCs (totally about 1 × 10^6^ cells) was added to the basal side of the MN arrays^16^. After a 30 s blue light curing process, the MN-MSC patch was obtained. The MN-MSC patch was cultured in MSC serum-free media for in vitro and in vivo testing. According to previous reports^22^, we estimated that 1 × 10^6^ cells could secrete approximately 1.68 × 10^10^ EV particles within the first week post-transplantation. Consequently, a roughly equivalent amount of MSC-EVs to the number of EVs secreted by MSCs was mixed into a 5% (w/v) porous GelMA solution, resulting in a final concentration of approximately 4.2 × 10^11^ particles/mL. Next, the 40 μL solution with EVs (totally 1.68 × 10^10^ EVs) was added to the basal side of the MN arrays at 37 °C. After a 30 s blue light curing process, the MN-MSC patch was obtained. The MN-MSC patch was cultured in MSC serum-free media for in vitro testing.

Gel-EVs were fabricated by direct photocuring a 5% (w/v) porous GelMA mixed MSC-EVs solution (40 μL solution, 1.68 × 10^10^ EVs) and Gel-MSCs fabricated by direct photocuring a 5% (w/v) porous GelMA mixed MSCs solution (40 μL solution, 1 × 10^6^ cells).

### MSCs viability analysis in porous MN-MSC Patch

The viability of MSCs in the MN-MSC patch was evaluated with a live/dead viability/cytotoxicity kit (L3224, Invitrogen, USA). After washing the MN-MSC patch with phosphate-buffered saline (PBS) for three times, the MSCs were stained with 0.2% ethidium homodimer-1(EthD) and 0.05% Calcein AM in PBS for 20 min. Then, the MN-MSC patch was washed with PBS for three times and observed by laser scanning confocal microscopy (Nikon, A1R). The proliferation of the MSCs in the MN-MSC patch was tested by CCK-8 assay (CK04, Dojindo, Japan) on the 1st, 3rd and 5th days. And the optical density of formazan was detected at a wavelength of 450 nm by a microplate reader (iD5, Molecular Devices, USA). To quantify the in vivo viability of MSCs in the porous MN-MSC patch, we implanted the MN-MSC patch in the injured spinal cord of rats using the surgical procedure described in the Animal and Surgical Procedures section. After implantation, we obtained images of the MSCs in the spinal cord injury site by H&E staining and immunofluorescence staining for GFAP/DAPI/hGAPDH. The images were acquired using a slide scanner (VS200, Olympus, Japan).

### Release and delivery analysis of the MN Patch

To compare the MSC secretome release capacity of MN-MSC patches fabricated with porous and traditional GelMA hydrogels, the amount of protein released from the patches was analyzed. Briefly, the porous MN-MSC patch and normal patch were stabilized in Transwells^52^, respectively (Fig. 4i). Next, the patches in 24-well plates containing MSC were cultured in serum-free media at 37 °C for two weeks, and the medium from the Transwell bottom was collected once every two days. The released proteins in the culture medium were quantified using a Micro BCA Protein Assay kit^53^. All release experiments were repeated three times for consistency and reliability of the results.

To evaluate the EV release capacity of the MN-MSC patch and MN-EV patch, we used a Transwell setup to simulate in vivo release (Fig. 2i). The immobilized patches were cultured in MSC serum-free media at 37 °C for two weeks, and we collected the medium from the Transwell bottom every two days. The released EVs were isolated from the collected medium using the differential centrifugation method described above and quantified by NTA (Marvin, UK, Nanosight NS500). We repeated each release experiment three times to ensure reliability and consistency of the results.

To quantify the EV delivery capacity of the MN patches, we used CM-DiI-labeled EVs (2 mg·mL^−1^, Yeasen, China) (CM-DiI-MN-EV) as a representative of the EVs released by MSCs. We implanted the CM-DiI-MN-EV patch in the injured spinal cord of rats using the surgical procedure described in the Animal and Surgical Procedures section. We obtained images of the stained EVs released in the spinal cord injury using a slide scanner (VS200, Olympus, Japan).

### Surgical Procedure

For in vivo experiments, a spinal contusion injury model was induced by an infinite vertical impactor (68099, RWD, China). To facilitate surgical procedures, we performed anesthesia for the contusive injury and transplantations using 1% (w/v) pentobarbital sodium following a well-established protocol to minimize rat mortality. Specifically, after being anesthetized, a laminectomy was performed at the tenth thoracic vertebral level (T10-11) of a rat to expose the dorsal surface to induce SCI. Next, contusion was performed using a cylinder (diameter, 3 mm) that impacted the spinal cord at a rate of 2.5 m/s and was left for 5 s at a depth of 2 mm after being impacted (Fig. S3b, c). To ensure blinding of the treatment conditions, a skilled technician who was independent and unaware of the treatment performed the implantation surgeries.

All implantation surgeries were performed by a technician who was blinded to the transplantation materials. An independent technician was responsible for randomly assigning rats, maintain treatment records, and label animals without involvement in the surgery. The implantation procedure involved three steps. First, the dura mater at the lesion site was cut carefully using a pair of microscissors (S11001-09, RWD, China) in the direction of the spinal cord. The second step is implanting. For MN, MN-EV, and MN-MSC group, the MN was placed at the site of the dura matter defect. 40 μL porous GelMA (5% (w/v)) (MN group) or GelMA (5% (w/v) contains 1.68 × 10^10^ particles EVs(MN-EV group) or MSCs (25,000 cells/μL,1 × 10^6^ cells, MN-MSCs group) were dripped onto the MN and irradiated with light at a wavelength of 405 nm for 1 min to create the patches (Fig. S3f). For Gel-EV and Gel-MSC group, the prepared Gel-MSC, Gel-EVs, were directly implanted on the site of the dura matter defect separately. For the MSC-Local group, MSCs were injected into the lesion (20 μl solution containing 1 × 10^6^ cells in PBS for each rat). Third, 5% (w/v) porous GelMA solution was added onto the surface of the exposed spinal cord with the patch and gelled with blue light to secure the patch on the spinal cord injury site (Fig. S3g–i). The control group and MSC-IV group rats underwent contusion and dura mater cutting without placement of any material. The MSC-IV group rats received a total of 1 × 10^6^ MSC injections at 3 day post-SCI following a reported protocol^16^. Additionally, MN-MSC group transplanted were conducted as the same method at 3, 7, 14 days after SCI. Moreover, MN-MSC + GW4689 group transplanted were conducted using the same method, but the 5% (w/v) porous GelMA containing GW4689 solution (20 μM)^44^. All rats were carefully sutured and labeled. Antibiotics (Baytril, 10 mg/kg) and painkillers (buprenorphine, 0.05 mg/kg) were administered to the animals for the first two weeks to ensure their well-being. The rats did not receive any immunosuppressive medication. Artificial micturition was performed twice daily until automatic bladder voiding was restored. To maintain surgical quality, no more than 15 rats were operated on per day.

At 6 weeks after injury, anterograde tracing of propriospinal axons was performed via the injection of AAV2/9-hSyn-mCherry at the T7-8 spinal cord by independent technician^33,35^. Specifically, a pull-glass micropipette tipped with a 10-μL Hamilton microsyringe (68606, RWD, China) was used for precise injection. After a laminectomy was performed at the T7-8 spinal cord, AAV2/9-hSyn-mCherry was injected into 12 sites at the following locations: (1) 0.4 and 0.8 mm lateral to the midline; (2) 0.5, 1.0 and 1.6 mm from the surface. The injection rate was 80 nl/min, and the injected liquid volume was 150 nl per site. The needle was left for 1 min before moving to the next. Animals were sacrificed for assessment 2 weeks after injection.

### Western blotting

At the post-SCI 7day, rats were randomized selected to assess their immunity. Another group of examiners blinded to the treatment groups processed the western blotting. Tissues were washed three times with ice-cold PBS and lysed in RIPA buffer containing 1% PMSF. After leaving the samples on ice for 30 min, they were centrifuged at 12,000*g* for 15 min at 4 °C. Protein concentrations in the supernatant were assessed using a Micro BCA Protein Assay kit. Equal amounts of protein extracts were resolved by 10–12% SDS-PAGE and electrotransferred onto a polyvinylidene membrane (Bio-Rad, USA). After blocking in Tris-buffered saline plus 5% (w/v) milk, the membranes were exposed to primary antibodies overnight at 4 °C. The samples were incubated with secondary antibodies conjugated to horseradish peroxidase for 1 h at room temperature. An independent examiner blinded to the treatment groups assessed these gene expressions. Signals were visualized by a ChemiDoc Touch Imaging System (Bio-Rad, USA), and ImageJ software was used to assess their average inflorescence intensity. All experiments were repeated three times.

### Implantation of electrodes

Electrode implantation was performed 8 weeks after SCI for both evoked potential and hindlimb EMG according to previously published protocols^33^. To minimize the effect on evoked potential recordings, we anesthetized the rats with dexmedetomidine hydrochloride (0.25 mg/kg i.p.; MCE, US) and 0.5% isoflurane^54^. A stainless steel screw electrode (the yellow star in Fig. 7b) was fixed on the skull above the hindlimb region stereotactically (Behind: −2.0 mm/ Right: +2.5 mm/ Deep: −0.7 mm compared with bregma (blue triangular in Fig. 7b)) for an epidural recording of cortical evoked potentials referring to the zero potential on the shoulder (ground lead (black circular in Fig. 7b)) connected to Behind: −6.0 mm/Right: 4.0 mm compared with bregma)^55^. Following the SSEP recording, bipolar electrodes (AS632, Conner wire) were inserted into the right medium gastrocnemius (GS) and TA for in vivo assessment of hindlimb muscles with the reference lead linked to the ipsilateral tendon^33^. Wires were subcutaneously connected to the header plate fixed on the skull. Electrode implantation was operated by an independent examiner blinded to the treatment groups.

### SSEP recording and processing

During the surgery, the header was connected to a neuron signal amplifier (BTAM01L, Braintech, China), and the signal was recorded by an independent examiner with a NeuroStudio system (Braintech, China)^47^. A series of pulses (2.0 Hz, 2.0 mA, 0.5 ms) was applied to the left hindlimb paw through a pair of electrodes by a neurostimulator (BTSEM-16, Braintech, China) under anesthesia^33^. Signals were obtained from a 5-min stimulation, followed by a 30- to 1500-Hz bandpass, power line noise notch filtration (50 Hz and the two consequent harmonics 100 Hz and 150 Hz), and the removal of stimulation artifacts. Then, the first 150 qualified trials were chosen for peak-to-peak potential (PPV) calculation. The PPV was normalized according to the intact group, following the method described by Gunnar Waterstraat et al.^56^.

### EMG recording and processing

Seven days post-implantation, five osteoarticular structures were captured to distinguish stance and swing when moving freely, and the header was connected up to the amplifier to detect muscle strength. Next, the free-moving muscle activity, represented by an extensor and a flexor of the right hindlimb, was logged in NeuroStudio. Data were filtered (bandpass 20 Hz-1000 Hz) before computing the muscle amplitude on the MATLAB platform^57^. The EMG Recording and Processing were by technicians blinded to the treatment groups. In this study, we employed the Poincaré analysis method to differentiate chaos from randomness by embedding datasets into a higher-dimensional state space. For instance, a time series of EMG peak signals was represented as follows:

$${{{x}}}_{{{t}}},{{{x}}}_{({{t}}+1)},{{{x}}}_{({{t}}+2)},\ldots,{{{x}}}_{({{t}}+{{n}})}$$

To generate a return map in its simplest form, we plotted (*x*_*t*_, *x*_(*t*+1)_), (*x*_(*t*+1)_, *x*_(*t*+2)_), and then (*x*_(*t*+2)_, *x*_(*t*+3)_), and so forth. *x*_*t*_ is the signal value of the electromyographic signal *x* at time *t*, where *t* = 1, 2,…, *t* + *n* are time points separated by n time intervals from time *t*, where *n* = 1, 2,…,. This approach allowed for a clear representation of the interval length between two EMG peak signals in a series, enabling rhythm quantification across different groups (Intact, control, MN, Gel-EV, Gel-MSC, MN-EV, and MN-MSC). Ultimately, we obtained both amplitude and rhythm information by analyzing the EMG data. In the Poincare plot, healthy individuals are represented by a flatter ellipse, whereas unhealthy individuals exhibit a larger length-to-width ratio, resulting in a wider ellipse in the Poincare plot.

### Histology

Animals were sacrificed at 8 weeks after injury for histological assessment. Independent examiners who were blinded to the treatment groups processed the spinal cord tissue and stained the slides. After the animals were anesthetized, perfusion was performed using PBS and 4% paraformaldehyde. Spinal cords and other tissues were fixed in 4% paraformaldehyde overnight and dehydrated with sucrose for 1 day. And the fixed tissues were dehydrated in 15 and 30% sucrose solutions. After being embedded using optimal cutting temperature compound (OCT), twenty-micrometer-thick sections of the spinal cord were cut using a cryostat (CryoStar NX50; Thermo, USA) and mounted onto slides. For sagittal continuous section analysis, we randomly selected several 8 mm long midsagittal sections which containing the epicenter of injury. And the examined sagittal sections of spinal cords were from the same level (T9-T11). For coronal section analysis, we selected rostral and caudal tissue from the region adjacent to the sagittal tissue. For immunofluorescence histochemistry, spinal cord tissue sections were blocked with 5% donkey serum and 0.3% Triton X-100 for 1 h and incubated with primary antibodies overnight at 4 °C. After washing with PBS three times, the tissues were incubated with secondary antibodies conjugated to fluorescent dyes for 2 h at room temperature. Then, tissues were rewashed with PBS and dripped with an antifade mounting medium containing DAPI. The sagittal serial sections were stained with 5-HT (serotonergic axons), RFP (propriospinal axons), NF (axonal fibers), GFAP (Astrocyte), NeuN (neurons), MBP (myelin sheath), CD31 (blood vessels), and Iba1 (microglia). The coronal serial sections were stained with 5-HT, RFP, and NF. Whole images were acquired using a slide scanner (VS200, Olympus, Japan) and for enlarged details, images were acquired using a confocal laser scanning microscope (A1Ti, Nikon, Japan). For the same staining images, all groups use the same imaging parameter. An independent technician who was blinded to the treatment group used the same region of interest to quantify fluorescent staining for these images. Specifically, the spared axons were quantified by their average fluorescence intensity in rostral and caudal sections, while microglia were quantified by Iba1 immunoreactivity average fluorescence intensity in sagittal sections. The area of other tissues, including those marked by GFAP, NeuN, MBP, and CD31, were quantified by their ratio to the total spinal cord area marked by DAPI in sagittal sections. At least three sections apart 40 μm were chosen for each spinal cord. All images adjust and fluorescence intensity quantification was using ImageJ software. After collecting all the data, the technician responsible for recording the treatments and animal tags divided the rats into different groups. The data from 3–5 animals were analyzed and organized into figures using Graphpad software.

Some spinal cord tissue and organ sections were stained with hematoxylin and eosin H&E. Images were acquired by a slide scanner (VS200, Olympus, Japan). For hematoxylin and eosin staining, spinal cord tissue and organ sections were hydrated in deionized water (DI) and left in hematoxylin for 5 min. The samples went through a series of washes and left in Eosin Y for 1 min and 20 s. The samples went through another series of ethanol washes and analyzed using slide scanner (VS200, Olympus, Japan).

### Behavioral assessment

The locomotion performance of the rats was recorded by an independent examiner with a camera once a week during the experiments. Another examiner blinded to the treatment assessed the videos recording the rats’ behavioral performance by the Basso, Beattie Bresnahan rating scale (BBB scale)^34^. Rats that showed a BBB score above 1.5 at 1 week after SCI were excluded from further histological or behavioral analysis. To precisely understand the locomotor kinematics, the representative hindlimb osteoarticular landmarks (toe, ankle, knee, hip, and crest) were labeled by 5 mm reflective balls and tracked by the MotoRater (Vicon Nexus, UK)^33^. Typical sequential treads and the ankle degrees of selected articulates were displayed. Body weight support and stride length were compared as previously described^35^.

The behavioral data were depicted with seven features (maximal iliac crest height; crest height amplitude; maximal toe height; toe height amplitude; and hip, knee and ankle angle oscillation) within four groups (intact, control, MN, Gel-EV, Gel-MSC, MN-EV, and MN-MSC). For each group, we utilized different statistical methods to evaluate the seven behavioral features, and these features have different units. As we needed to thoroughly analyze these features in radar graphs, normalizing these data was vital and pivotal. First and foremost, for each value in each feature, we normalized the behavioral data of the seven features by

1 $$\widetilde{{x}_{i}}=\frac{\left({x}_{i}-\bar{x}\right)}{\sqrt{\sigma }}$$

where *x*_*i*_ represents the *i*th data point for each of the seven behavioral features, *I* = 1, 2, 3,...,*n*. *n* is the number of points sampled from each feature in all groups. $\bar{x}$ and $\sqrt{\sigma }$ represent the mean value and standard variance for each feature, respectively.

Second, we obtained the seven $\widetilde{{x}_{i}}$ values. Each group had positive and negative values, and the scale among these values was not fixed. Hence, we projected the features into an exponentiation expression, mapping these values in a unified scale (a unified vector space). For each feature depicted in a different group, we used equation (2) to process this step:

2 $$\widetilde{{x}_{f}}=\frac{{{{{{{\mathrm{average}}}}}}}({e}^{\widetilde{{x}_{i}}})}{\max ({{{{{{{\mathrm{average}}}}}}}}({e}^{\widetilde{{x}_{i}}}))}$$

where *f* = 1, 2, 3,…,7, represents the seven behavioral features in each group. We averaged the ${e}^{\widetilde{{x}_{i}}}$ in each group, obtaining lists with seven feature values in each group.

In the final analysis, we used the intact group values as the benchmark, significantly depicting the feature values’ variance. We used equation (3) to process this step:

3 $${\widetilde{x}}_{o}=\frac{{\widetilde{x}}_{g}}{{\widetilde{x}}_{{{{{{{\rm{intact}}}}}}}}}$$

where ${\widetilde{x}}_{g}$ represents each feature value in each group. ${\widetilde{x}}_{{{{{{{\rm{intact}}}}}}}}$ represents the feature values in the intact group, namely, the benchmark values.

### Statistics

Multiple samples were analyzed by one-way or two-way analysis of variance (ANOVA). In this study, we used one-way ANOVA to compare data with only one factor (e.g., treatment) and two-way ANOVA to compare data with two factors (e.g., treatment and time). Differences between the two experimental group (e.g. control and MN-MSC patch) were analyzed by Student’s *t* test. All parameters were expressed as the mean ± standard error of the mean (SEM), and a P value less than 0.05 was considered significant. Graphpad software (v8.3.0, USA) and Python package (SciPy, v1.4.1) were used for statistical analysis and visualization. Electromyography (EMG) data analysis and behavioral assessment were using MATLAB(v2020b). For microscopy, images were analyzed using Fiji (Windows 64, v1.51, NIH).

### Reporting summary

Further information on research design is available in the Nature Portfolio Reporting Summary linked to this article.

## Supplementary information

Supplementary information

Description of additional supplementary files

Supplementary Movie 1

Reporting Summary

## Data Availability

The data to support the findings of this study are included in the paper and supplementary information. The raw data for figures were provided in the supplementary source data file. Any additional requests for information can be directed to, and will be fulfilled by, the lead contact. Source data are provided with this paper.

## Source data

Source data

## Code availability

All figures were directly generated in MATLAB and Python from the code available at the GitHub repository at https://github.com/StarLight1212/NC_proj^58^. The data used and generated in this study can be found at https://drive.google.com/drive/folders/1az7uOLzP5xH7K-zpFzoAkv36kasVc-AW.
