# Author Correction: Porous microneedle patch with sustained delivery of extracellular vesicles mitigates severe spinal cord injury

**DOI:** 10.1038/s41467-023-40368-w

**Published:** 2023-08-01

**Authors:** Ao Fang, Yifan Wang, Naiyu Guan, Yanming Zuo, Lingmin Lin, Binjie Guo, Aisheng Mo, Yile Wu, Xurong Lin, Wanxiong Cai, Xiangfeng Chen, Jingjia Ye, Zeinab Abdelrahman, Xiaodan Li, Hanyu Zheng, Zhonghan Wu, Shuang Jin, Kan Xu, Yan Huang, Xiaosong Gu, Bin Yu, Xuhua Wang

**Affiliations:** 1 Department of Rehabilitation Medicine of First Affiliated Hospital and School of Brain Science and Brain Medicine, Zhejiang University School of Medicine, 310003 Hangzhou, Zhejiang PR China; 2 Liangzhu Laboratory, MOE Frontier Science Center for Brain Science and Brain-machine Integration, State Key Laboratory of Brain-machine Intelligence, Zhejiang University, 1369 West Wenyi Road, 311121 Hangzhou, PR China; 3 NHC and CAMS Key Laboratory of Medical Neurobiology, Zhejiang University, 310058 Hangzhou, PR China; 4 Department of Orthopedics of 2nd Affiliated Hospital and School of Brain Science and Brain Medicine, Zhejiang University School of Medicine, Zhejiang University, 310003 Hangzhou, Zhejiang PR China; 5 Department of Hepatobiliary and Pancreatic Surgery, Affiliated Hospital of Nantong University, Medical School of Nantong University, 226001 Nantong, PR China; 6 Key Laboratory of Neuroregeneration of Jiangsu and Ministry of Education, NMPA Key Laboratory for Research and Evaluation of Tissue Engineering Technology Products, Nantong University, Nantong, PR China; 7 Co-Innovation Center of Neuroregeneration, Nantong University, 226001 Nantong, Jiangsu PR China

**Keywords:** Spinal cord injury, Biomedical materials, Mesenchymal stem cells, Biomedical engineering, Biomaterials - cells

Correction to: *Nature Communications* 10.1038/s41467-023-39745-2, published online 07 July 2023

The original version of this Article contained an error in the ‘The MN-MSC patch alleviates the inflammatory response in spinal cord lesions after SCI’ section of the Results, which incorrectly read ‘However, only the MN- EV treatment yielded a statistically significant increase in the anti-inflammatory cytokine TGF-β as well as increased arginase-2 and decreased IL-1β expression.’ The correct version states ‘However, only the MN-MSC treatment yielded a statistically significant increase in the anti-inflammatory cytokine TGF-β as well as increased arginase-2 and decreased IL-1β expression.’ This has been corrected in both the PDF and HTML versions of the Article.

