# Supplementary material for: Porous microneedle patch with sustained delivery of extracellular vesicles mitigates severe spinal cord injury
Source: Nat Commun. 2023 Jul 7;14:4011. doi: 10.1038/s41467-023-39745-2 (PMC10328956; doi:10.1038/s41467-023-39745-2)
Supplement: Supplementary file 1 — Supplementary information [file 41467_2023_39745_MOESM1_ESM.pdf]

Supplementary Information for "Porous microneedle patch with sustained delivery of extracellular vesicles mitigates severe spinal cord injury"

Ao Fang[1,2,3,4,8], Yifan Wang[1,4,8], Naiyu Guan[1,2,3,4,8], Yanming Zuo[1,2,3,8], Lingmin Lin[2,3], Binjie Guo[2,3], Aisheng Mo[2,3], Yile Wu[2,3], Xurong Lin[2,3], Wanxiong Cai[2,3], Xiangfeng Chen[2,3,4], Jingjia Ye[2,3,4] , Zeinab Abdelrahman[1,2,3,4], Xiaodan Li[1,2,3,4], Hanyu Zheng[2,3], Zhonghan Wu[2,3], Shuang Jin[2,3], Kan Xu[4], Yan Huang[5], Xiaosong Gu[6], Bin Yu[6], Xuhua Wang[1,2,3,4,7]

Affiliations:

1. Department of Rehabilitation Medicine of First Affiliated Hospital and School of Brain Science and Brain Medicine, Zhejiang University School of Medicine, Hangzhou, Zhejiang Province 310003, P. R. China
2. Liangzhu Laboratory, MOE Frontier Science Center for Brain Science and Brain-machine Integration, State Key Laboratory of Brain-machine Intelligence, Zhejiang University, 1369 West Wenyi Road, Hangzhou 311121, China
3. NHC and CAMS Key Laboratory of Medical Neurobiology, Zhejiang University, Hangzhou 310058, China
4. Department of Orthopedics of 2nd Affiliated Hospital and School of Brain Science and Brain Medicine, Zhejiang University School of Medicine, Zhejiang University, Hangzhou, Zhejiang Province, 310003, PR China
5. Department of Hepatobiliary and Pancreatic Surgery, Affiliated Hospital of Nantong University, Medical School of Nantong University, Nantong, 226001, China
6. Key Laboratory of Neuroregeneration of Jiangsu and Ministry of Education, NMPA Key Laboratory for Research and Evaluation of Tissue Engineering Technology Products, Nantong University, Nantong, China
7. Co-innovation Center of Neuroregeneration, Nantong University, Nantong, 226001 Jiangsu, PR China
8. These authors contributed equally

Correspondence should be addressed to X.W. (email: xhw@zju.edu.cn).

Supplementary table 1. Antibodies used in this study.

Reagents	Source	Identifier	Applied ratio
Chicken anti-GFAP	Abcam	ab134436	1:500
GAPDH (Human Specific) Rabbit mAb	Abclonal	AC036	1:500
rabbit anti-neurofilament (NF) heavy polypeptide	Abcam	ab8135	1:500
goat anti 5-HT antibody	abcam	ab66047	1:500
rabbit anti-NeuN	Abcam	ab177487	1:1000
Rabbit polyclonal to RFP	Abcam	ab62341	1:500
rabbit anti-MBP	Abcam	ab218011	1:500
goat anti-IBA1	Abcam	ab5076	1:500
rabbit anti-CD31	R&Drd system	AF3628	1:500
GAPDH Rabbit Monoclonal Antibody	Beyotime	AF1186	1:1000
rabbit anti-TGF-β	Abclonal	A2124	1:1000
rabbit anti-BAX	Abclonal	A0207	1:1000
rabbit anti-MMP-9	Abclonal	A0289	1:1000
rabbit anti-Arginase 2 (Arg-2)	Abclonal	A19233	1:1000
Donkey anti-Chicken IgY H&L (FITC)	Abcam	ab63507	1:500
Donkey anti-Rabbit IgG H&L (Alexa Fluor® 555)	Abcam	ab150062	1:500
Rabbit anti-Goat IgG H&L (Alexa Fluor® 555)	Abcam	ab150142	1:500
Donkey anti-Rabbit secondary antibodies (HRP)	beyotime	A0208	1:1000
Rabbit anti-iNOS	proteintech	80517-1-RR	1:2000
Rabbit anti-TNF-α	proteintech	17590-1-AP	1:1000
Rabbit anti-IL-1β	Abclonal	A20527	1:1000
Rabbit α-Tubulin	beyotime	AG0126	1:1000
PE Mouse anti-Human CD105	BD Pharmingen™	560839	1:20
APC Mouse Anti-Human CD90	BD Pharmingen™	561971	1:20

PE Mouse Anti-Human CD73	BD Pharmingen™	550257	1:20
FITC Mouse Anti-Human CD45	BD Pharmingen™	561865	1:20
FITC Mouse Anti-Human CD34	BD Pharmingen™	560942	1:20
APC Mouse Anti-Human CD19	BD Pharmingen™	561742	1:20
APC Mouse Anti-Human CD14	BD Pharmingen™	555399	1:20
PE Mouse Anti-Human CD11b	BD Pharmingen™	555388	1:20
PE Mouse Anti-Human CD79a	BD Pharmingen™	561942	1:20
PE Mouse Anti-Human HLA-DR	BD Pharmingen™	555812	1:20

Supplementary table 2. The experimental groups in this study.

Groups	MSC injection	MSC	Micron-eedle Patch	GelMA gel block	MSC -EVs	Notes
SCI only Control						SCI only control
MN-EVs			X	X	X	Microneedle Patch with GelMA gel block with MSC-EVs control
MN-MSC		X	X	X		Microneedle Patch with GelMA gel block with MSC
Gel-EVs				X	X	GelMA gel block with MSC-EVs control
Gel-MSC		X		X		GelMA gel block with MSC control
MN			X			Microneedle Patch alone control
MSC-Local	X (Local)					Local injection of MSC
MSC-IV	X (IV)					Intravenous administration of MSC control
MN-MSC+GW4869		X (EVs blockage)	X	X		Only soluble protein control

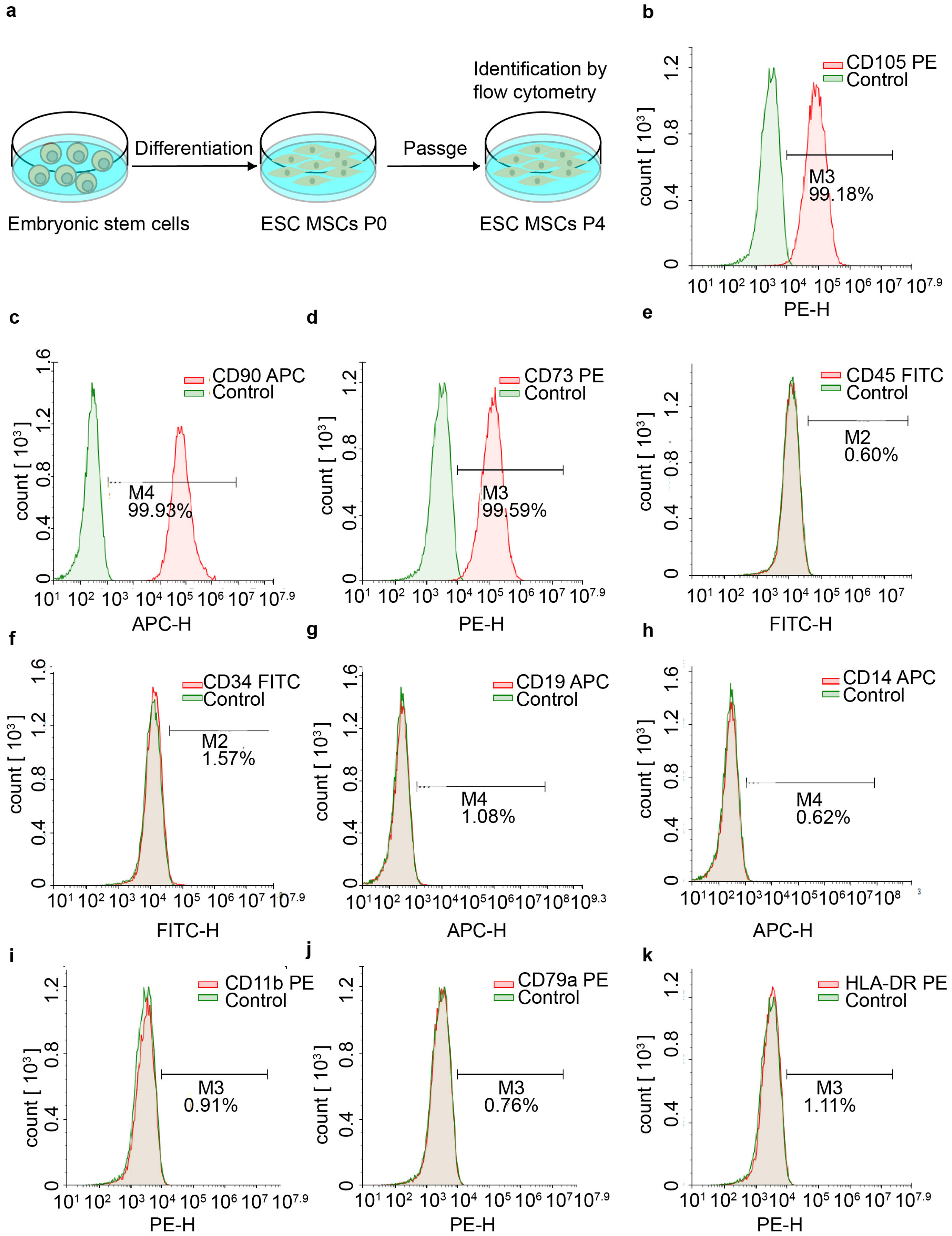

Fig.S1|Acquisition and identification of ESC-MSCs. a Acquisition steps of ESC-MSCs. **b-k** The expression of MSC-specific surface markers was analyzed by flow cytometry: **b** CD105 protein; **c**

CD90 protein; **d** CD73 protein; **e** CD45 protein; **f** CD34 protein; **g** CD19 protein; **h** CD14 protein; **i** CD11b protein; **j** CD79a protein; and **k** HLA-DR protein.

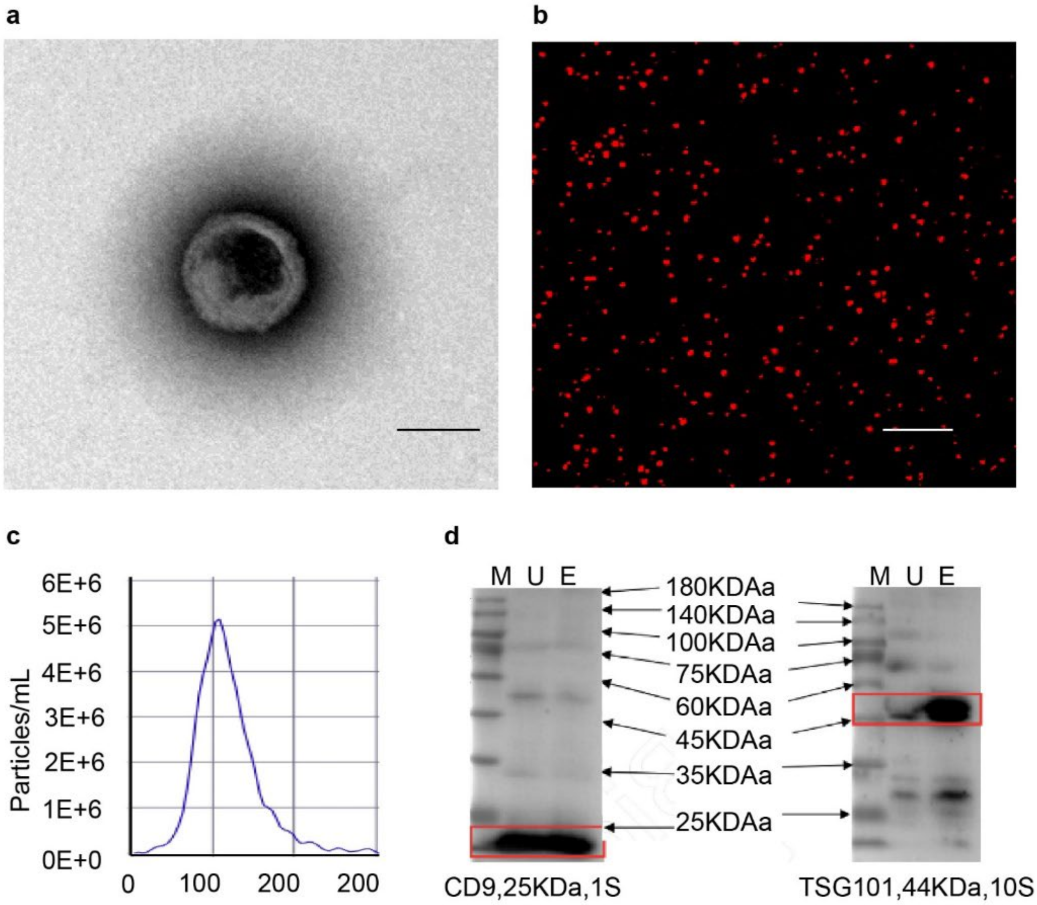

Fig. S2| Characterization of exosomes excreted by MSCs. **a** Morphology of exosomes revealed by TEM. Scale bar: 100 nm. **b** Exosomes labeled by CM-DiI (red) embedded in GelMA hydrogel. Scale bar: 50 μm. The experiment was repeated 3 times independently with similar results. **c** Particle size distribution as measured by NTA. **d** Expression of the membrane surface proteins CD9 and TSG101 detected by Western blot analysis. The experiment was repeated 3 times independently with similar results.

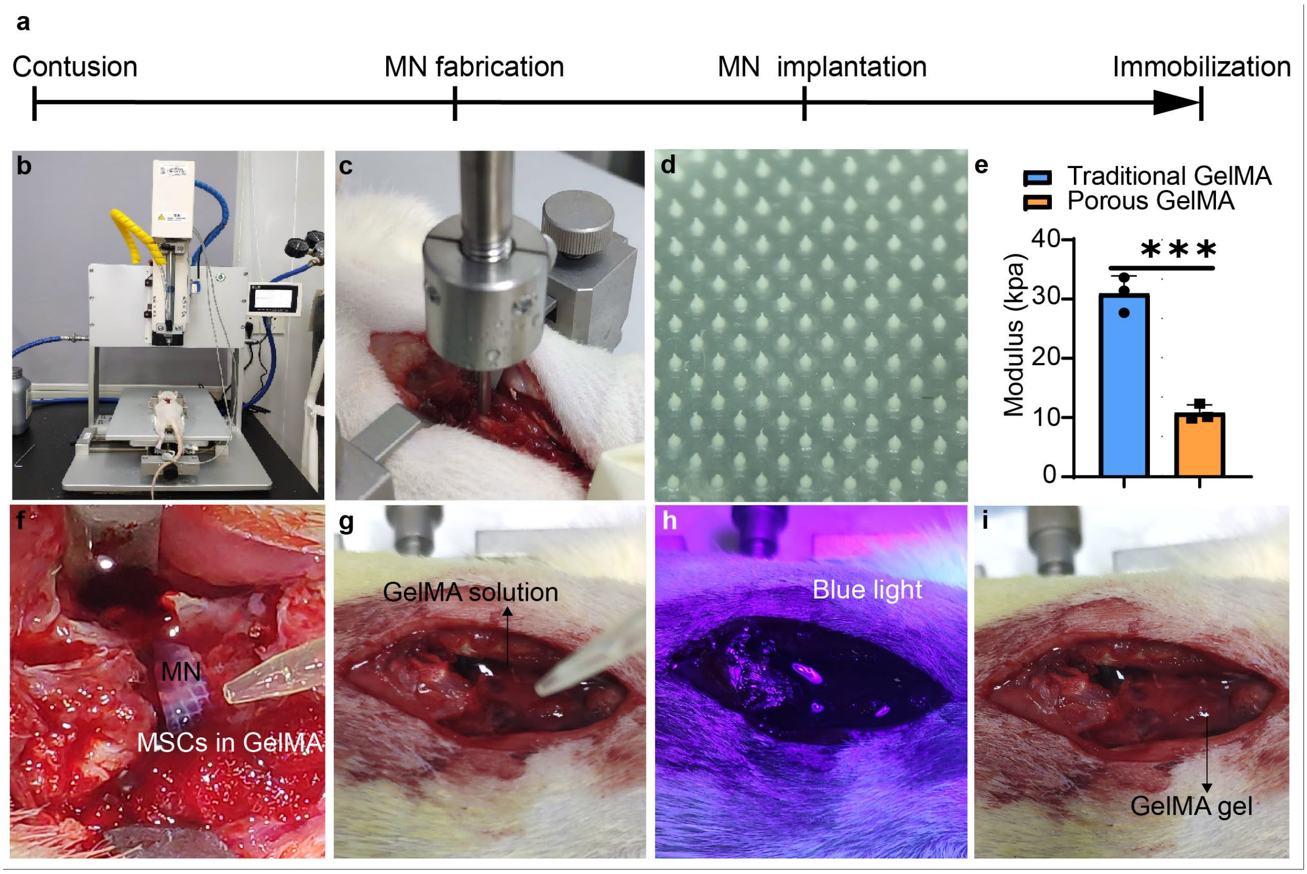

Fig. S3|The procedures for implanting the MN-MSC patch to treat a rat with SCI. a Schematic illustration of surgical steps. **b** Infinite vertical impactor. **c** SCI construction. **d** Fabricated MN array. **e** Mechanical strength of MN. n = 3 samples from each group. Data are presented as the mean ± SEM. Two-tailed paired t-tests were used for comparisons between two groups, p= 0.0005, ***p < 0.001. **f** Implantation of the MN array after dura removal. **g** GelMA solution was added. **h** Blue light crosslinking of GelMA on the SCI site. **i** The encapsulation of the MN-MSC patch and SCI with gelled GelMA.

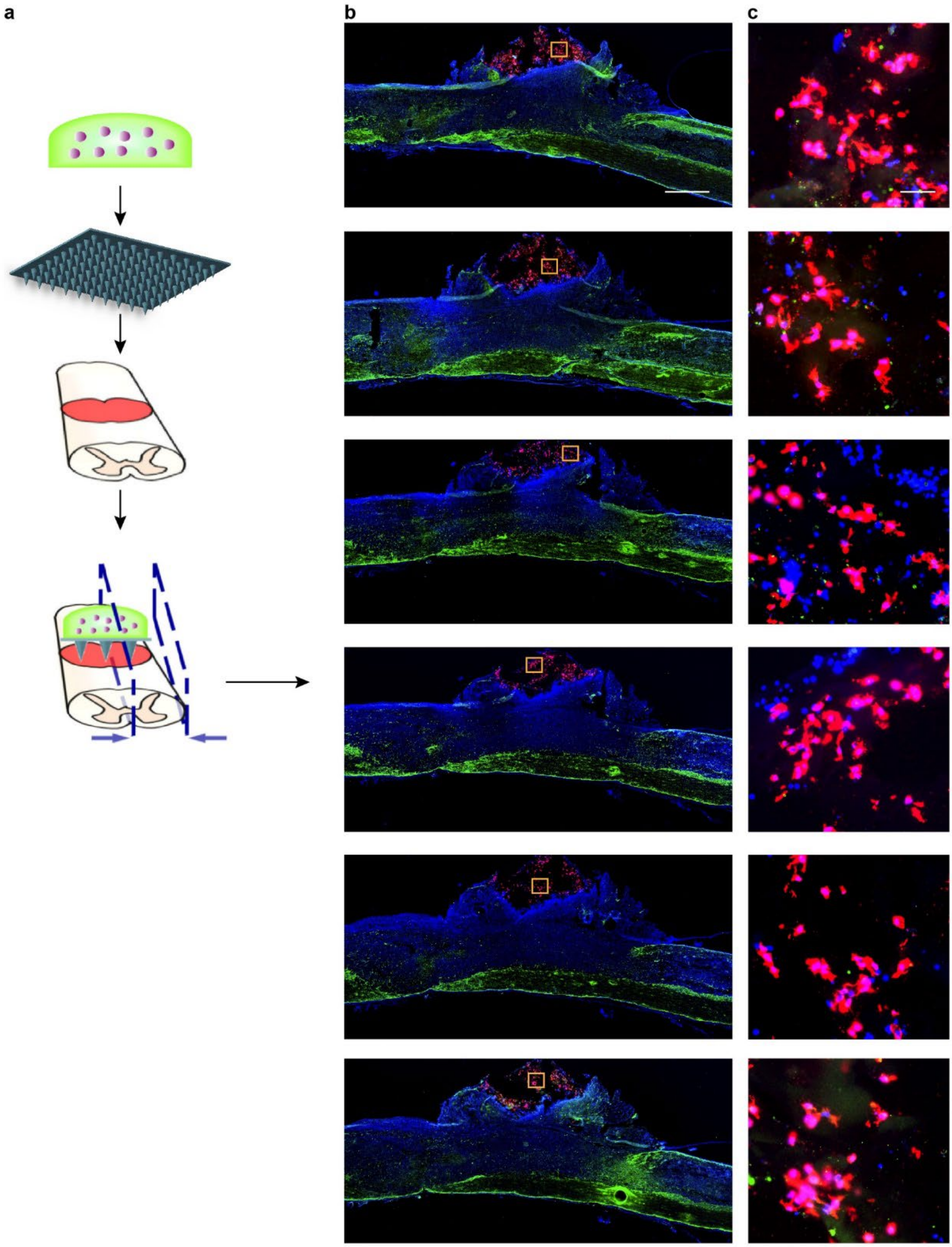

Fig. S4| Representative serial images of sagittal sections of the spinal cord of a rat treated with MN-MSC patch, green (GFAP), blue (DAPI), and red (GAPDH). a Schematic illustration of injured spinal cord treated with an MN-MSC patch. **b** Serial images of sagittal sections of different positions from the border to the center. Scale bar: 1 mm. The experiment was repeated 3 times independently with similar results. **c** Show details of MSCs encapsulated in the MN-MSC patch. Scale bar: 50 μm. The experiment was repeated 3 times independently with similar results.

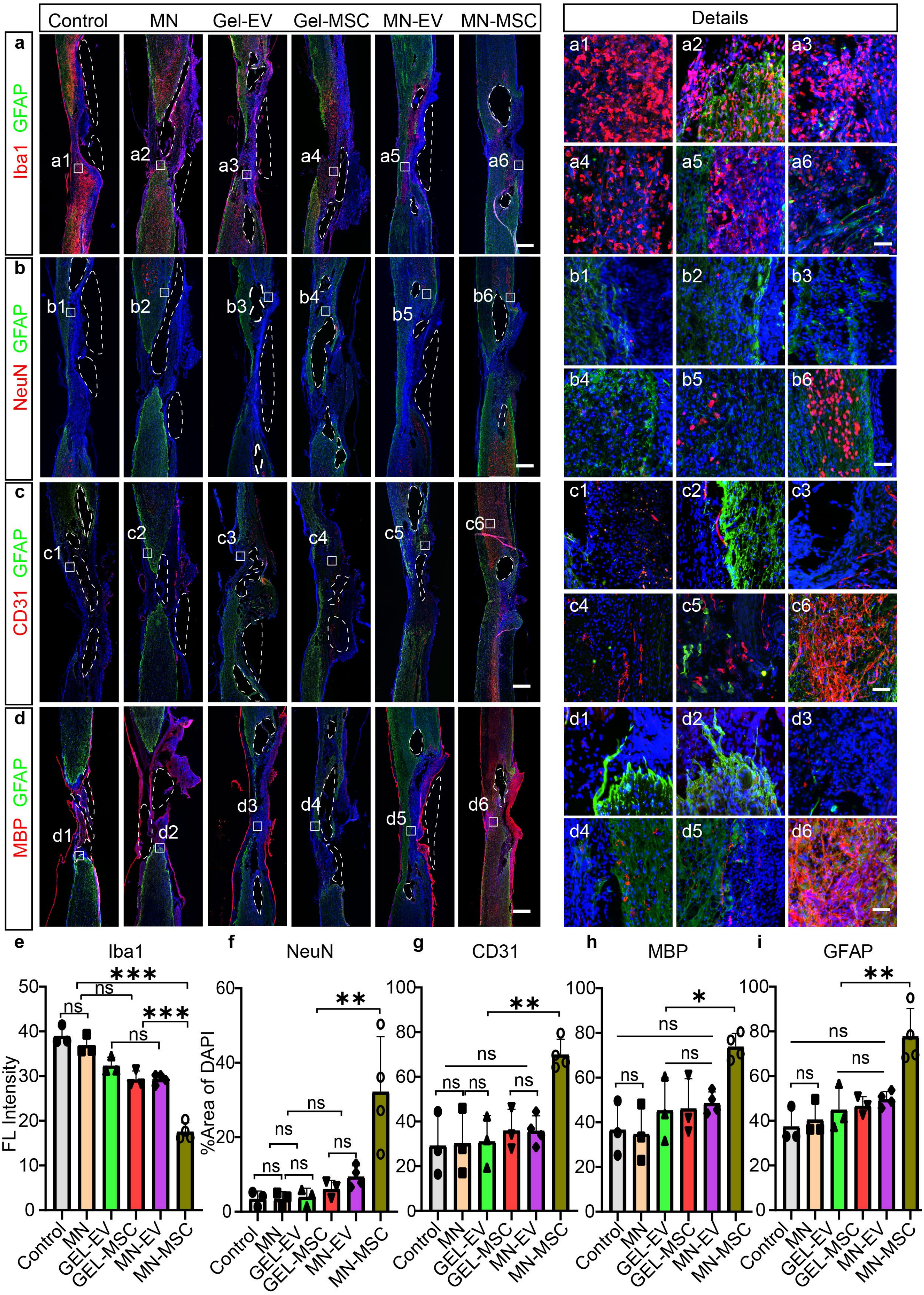

Control
MN
Gel-EV
Gel-MSC
MN-EV
MN-MSC
Details
a
Iba1 GFAP
b
NeuN GFAP
c
CD31 GFAP
d
MBP GFAP
e
Iba1
FL Intensity
f
NeuN
%Area of DAPI
g
CD31
h
MBP
i
GFAP
Control
MN
GEL-EV
GEL-MSC
MN-EV
MN-MSC

Fig. S5| Representative images of immunohistochemical staining of spinal cord sections. a Iba1, **b** NeuN, **c** CD31, and **d** MBP (red) in the six groups (control, MN, Gel-EV, Gel-MSC, MN-EV, and MN-MSC) at 8 weeks after injury. Scale bars for images **a-d** indicate 1 mm, while a1-a6, b1-b6, c1-c6, and d1-d6 indicate 100 μm. **e** Quantification of average fluorescence intensity of Iba1. Control: n=3, MN: n=3, Gel-EV: n=3, Gel-MSC: n=3, MN-EV: n=4, and MN-MSC: n=4 animals chosen randomly from each group. ANOVA for Iba1: Total: F= 61.06, p<0.0001, MN vs MN-MSC, p <0.0001. MN-EV vs MN-MSC, p <0.0001. *p < 0.05, **p < 0.01, ***p < 0.001. Data are presented as the mean ± SEM. Statistical analysis was performed using one-way ANOVA followed by Tukey's multiple comparisons test and two-tailed paired t-tests were used for comparisons between two groups. **f-i** area ratio of NeuN (f), CD31 (g), MBP (h), and GFAP (i) in total spinal cord area (marked by DAPI) in the injured spinal cord of the six groups. Data are presented as the mean ± SEM. Statistical analysis was performed using one-way ANOVA followed by Tukey's multiple comparisons test and two-tailed paired t-tests were used for comparisons between two groups. Control: n=3, MN: n=3, Gel-EV: n=3, Gel-MSC: n=3, MN-EV: n=4, and MN-MSC: n=4 animals chosen randomly from each group. ANOVA for NeuN: Total: F= 9.364, p= 0.0004, MN-EV vs MN-MSC, p= 0.0051. ANOVA for CD31: Total: F= 8.348, p= 0.0008, MN-EV vs MN-MSC, p= 0.0044. ANOVA for MBP: Total: F= 6.461, p= 0.0026, MN-EV vs MN-MSC, p= 0.0418. ANOVA for GFAP: Total: F= 11.33, p= 0.0002, MN-EV vs MN-MSC, p= 0.0032. *p < 0.05, **p < 0.01, ***p < 0.001.

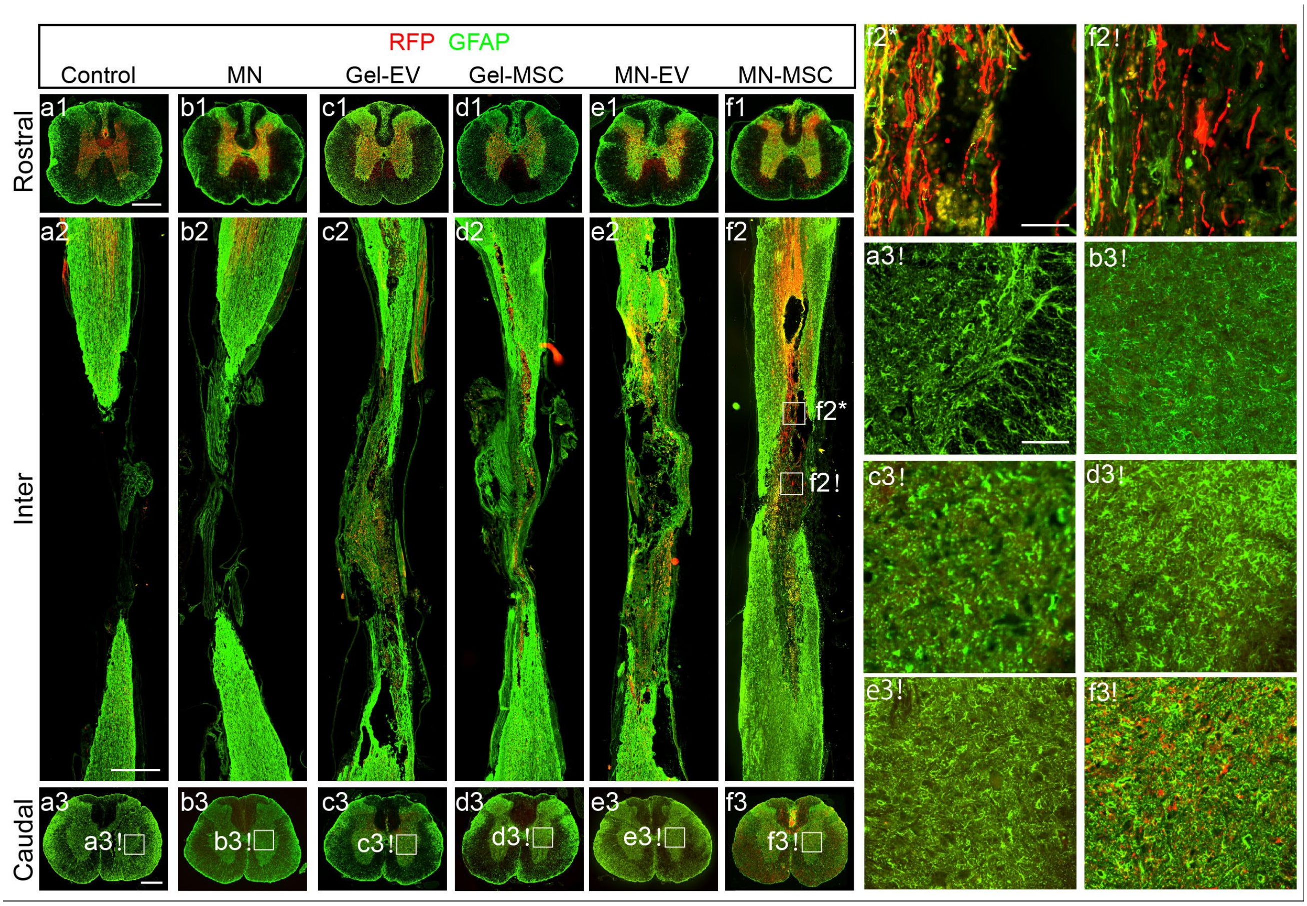

Fig. S6| related to Fig.5 Representative images of spinal cord sections stained with GFAP (green) and RFP (red) in the six groups (control, MN, Gel-EV, Gel-MSC, MN-EV, and MN-MSC). Scale bars: 1 mm (inter), and 500 μm (rostral, caudal). Detailed images in the white box shown in a3, b3, c3, d3, e3, f2, f3, named as a3!-f3!, f2* and f2! Scale bars: 100 μm.

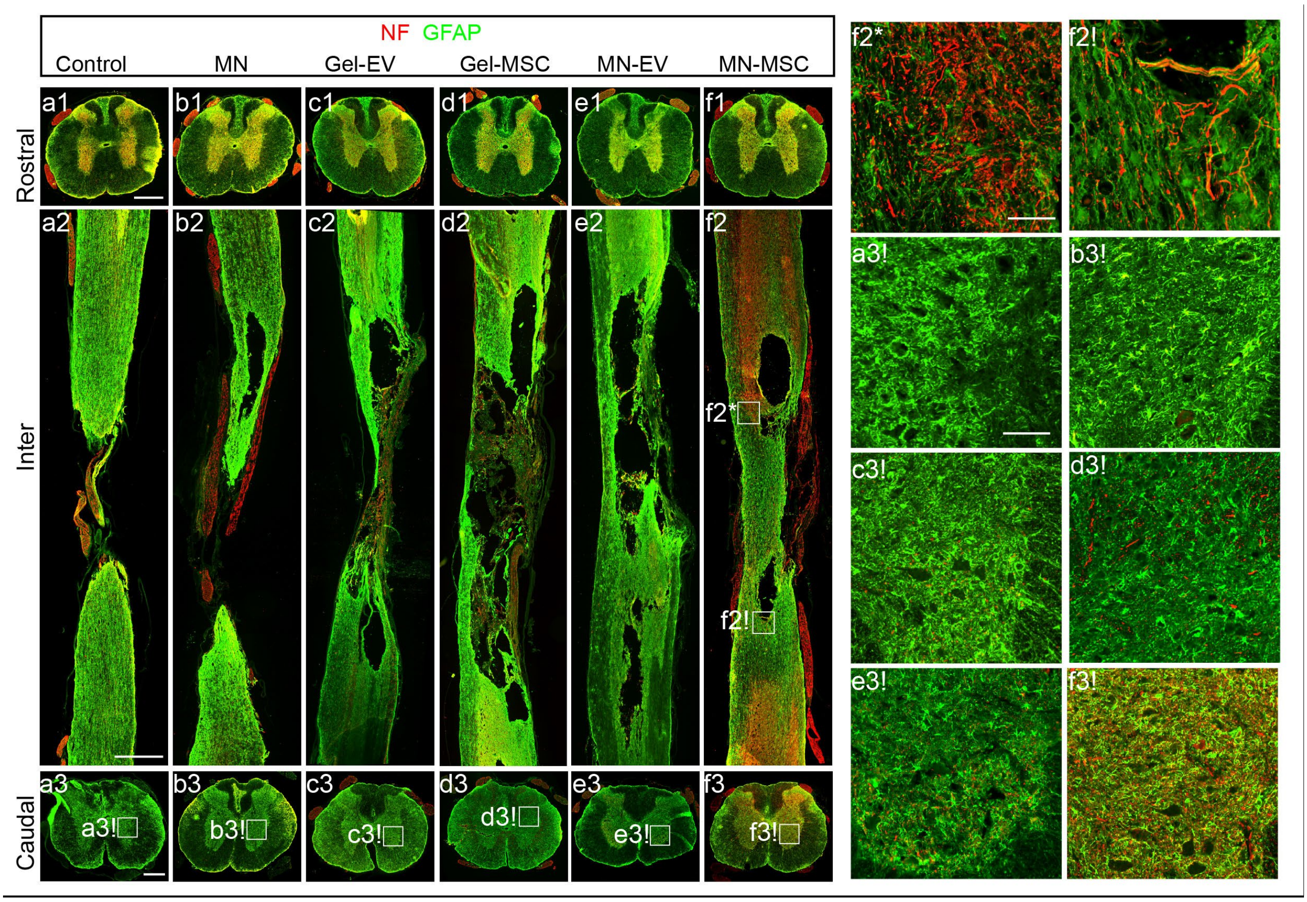

Fig. S7| Related to Fig. 5, **Representative images of spinal cord sections stained with GFAP (green) and NF (red) in rats from six groups (control, MN, Gel-EV, Gel-MSC, MN-EV, and MN-MSC).** Scale bars: 1 mm (inter), and 500 μm (rostral, caudal). Detailed images in the white box shown in a3, b3, c3, d3, e3, f2, f3, named as a3!-f3!, f2* and f2! Scale bars: 100 μm.

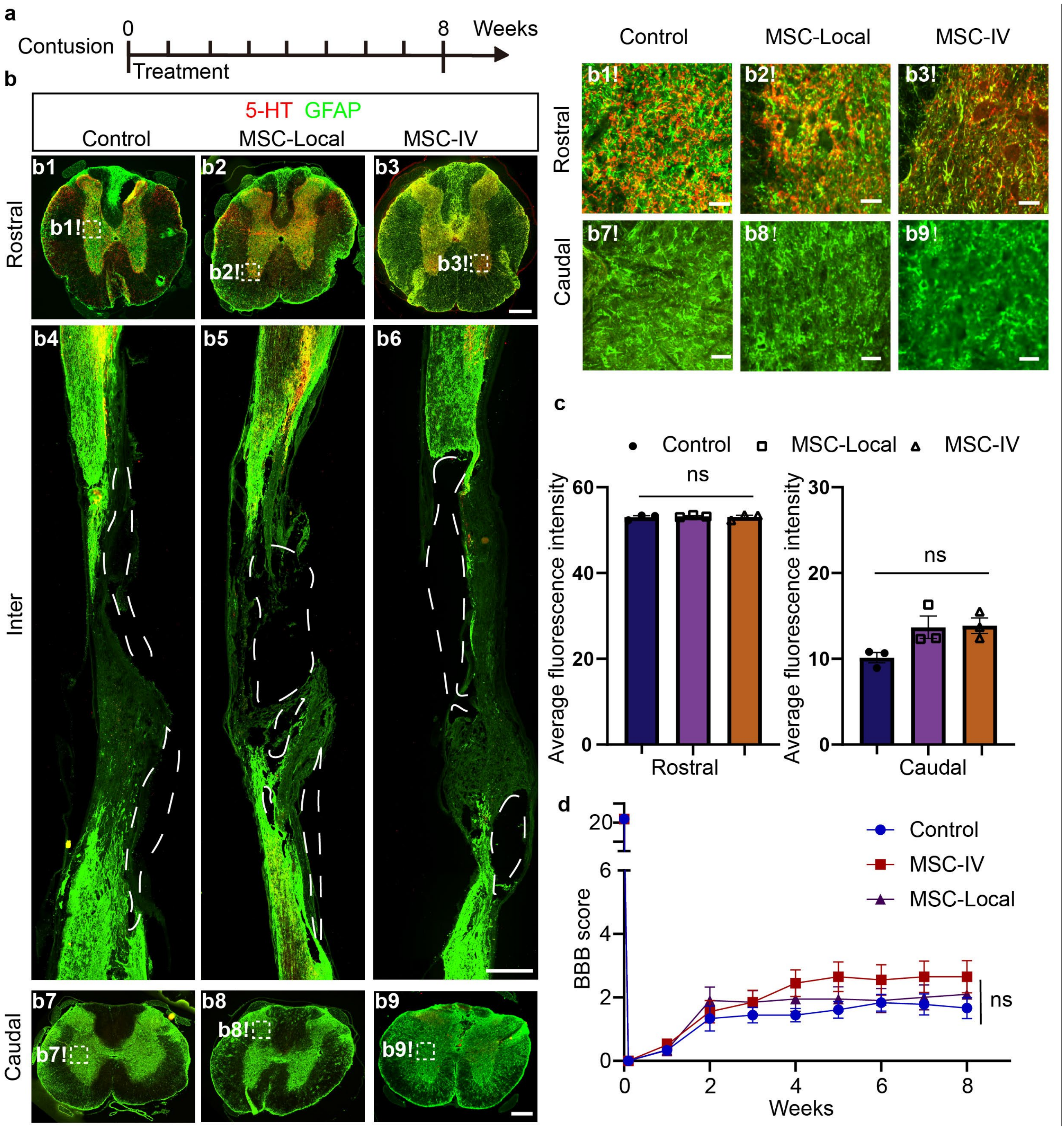

Fig. S8| Detailed analysis of rats with MSC-Local and MSC-IV treatments a Schematic illustration of this experiments. **b** Representative images of spinal sections stained with GFAP (green) and 5-HT (red) from rats in the three groups (Control, MSC-Local, MSC-IV). Scale bars: 1 mm, 200 μm (rostral and caudal sections). Detailed information in the white box in b1!-b3! and b7!-b9!. Scale bars: 40 μm (b1!-b3! & b7!-b9!)**. c** Quantification of the average fluorescence intensity of 5-HT immunoreactivity at the same segment of rats' spinal cord from three groups on the rostral and caudal sides, respectively. Data are shown as the mean ± SEM. Statistical analysis was performed using one-way ANOVA followed by Tukey's multiple comparisons test and two-tailed paired t-tests were used for comparisons between two groups. n=3 animals chosen randomly from each group. ANOVA for rostral: Total: F= 0.1304, p= 0.8802. ANOVA for caudal: Total: F= 4.553, p= 0.0627. **d** Weekly comparison of BBB scores among rats in the three groups (Control, MSC-Local, MSC-IV) at 8 weeks after SCI. Two-way ANOVA with Tukey's post-hoc test was used for comparisons among multiple groups, and two-tailed paired t-tests were used for comparisons between two groups. Total: F=0.6204, p= 0.8968. Control: n=9, MSC-Local: n=10, MSC-IV: n=10 independent animals for each group.

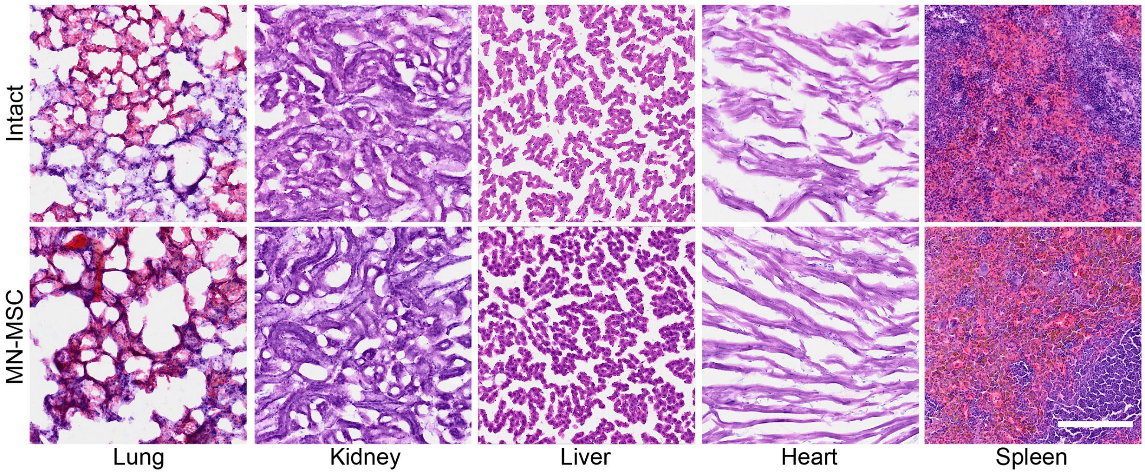

Fig. S9| H&E staining of the main organs of rats in the intact and MN-MSC groups. Scale bar: 200 μm. The experiment was repeated 3 times independently with similar results.

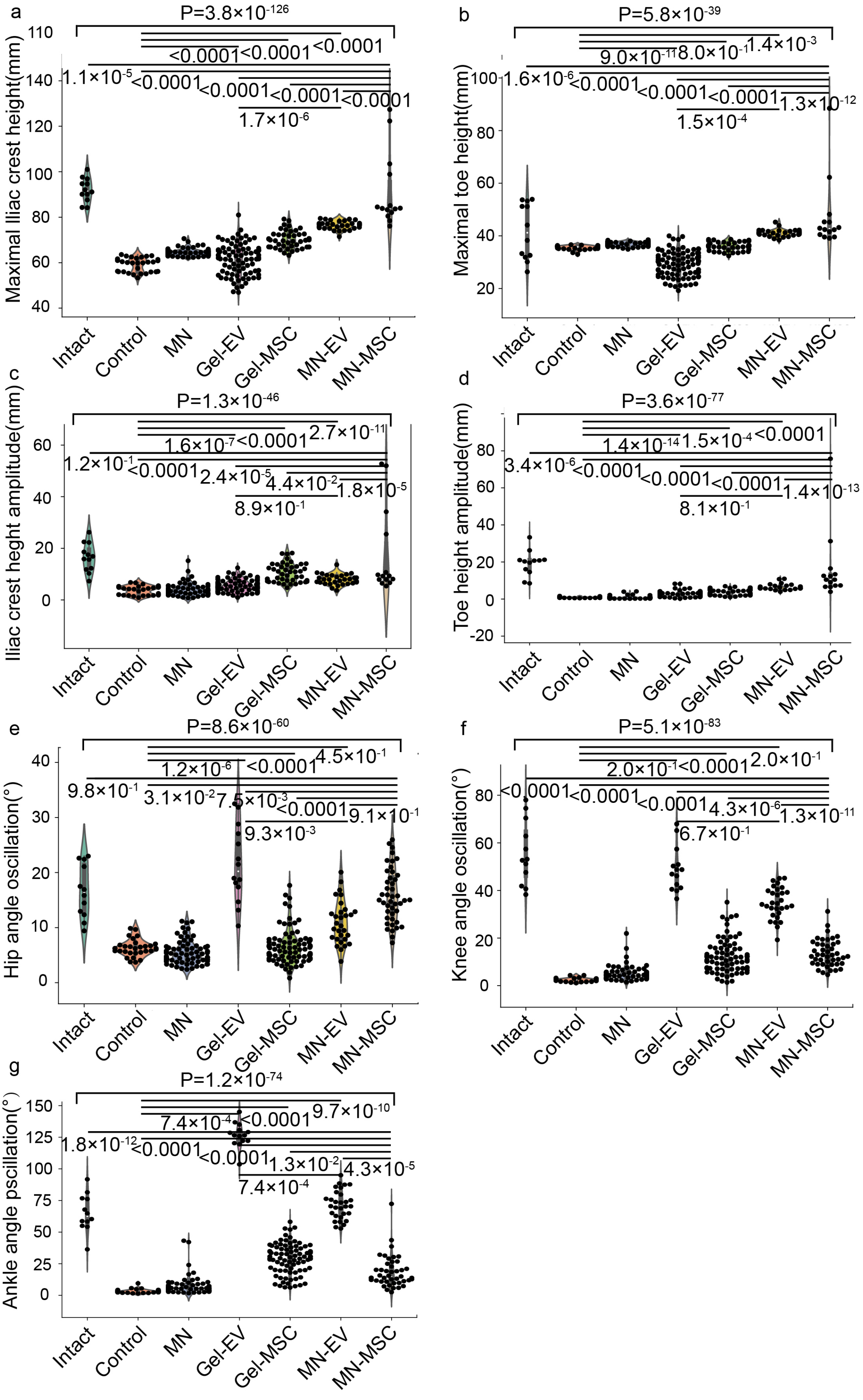

a
P=3.8×10-126
Maximal Iliac crest height(mm)
<0.0001 <0.0001 <0.0001
1.1×10-5 <0.0001 <0.0001 <0.0001 <0.0001
1.7×10-6
b
P=5.8×10-39
Maximal toe height(mm)
9.0×10-11 8.0×10-1 1.4×10-3
1.6×10-6 <0.0001 <0.0001 <0.0001 1.3×10-12
1.5×10-4
c
P=1.3×10-46
Iliac crest heght amplitude(mm)
1.6×10-7 <0.0001 2.7×10-11
1.2×10-1 <0.0001 2.4×10-5 4.4×10-2 1.8×10-5
8.9×10-1
d
P=3.6×10-77
Toe height amplitude(mm)
1.4×10-14 1.5×10-4 <0.0001
3.4×10-6 <0.0001 <0.0001 <0.0001 1.4×10-13
8.1×10-1
e
P=8.6×10-60
Hip angle oscillation(°)
1.2×10-6 <0.0001 4.5×10-1
9.8×10-1 3.1×10-2 7.5×10-3 <0.0001 9.1×10-1
9.3×10-3
f
P=5.1×10-83
Knee angle oscillation(°)
2.0×10-1 <0.0001 2.0×10-1
<0.0001 <0.0001 <0.0001 4.3×10-6 1.3×10-11
6.7×10-1
g
P=1.2×10-74
Ankle angle pscillation(°)
7.4×10-4 <0.0001 9.7×10-10
1.8×10-12 <0.0001 <0.0001 1.3×10-2 4.3×10-5
7.4×10-4
Intact
Control
MN
Gel-EV
Gel-MSC
MN-EV
MN-MSC

Fig. S10| Related to Fig. 6. Detailed statistical analysis of the hindlimb movement of rats in different groups. Quantification of a) the average maximal iliac crest height, b) maximal toe height, c) iliac crest height amplitude, d) toe height amplitude, e) hip angle oscillation, f) knee angle oscillation, and g) ankle angle oscillation in the different groups. One-way ANOVA with Tukey's post-hoc test for comparisons among multiple groups (*) and two-tailed paired t tests were used for comparisons within groups (#) for the data shown in the violin plot. The violin plot center indicates the median in all planes. Violin range covers 97.5th and 2.5th percentiles; extending whiskers show data distribution and probability density. Violin areas remain constant. Boxplot centerlines signify medians; boxes show first and third quartiles (Q1, Q3); whiskers extend from Q1 - 1.5xIQR to Q3 + 1.5xIQR; outliers lie outside whiskers. Intact: n=12, Control: n=31, MN: n=72, Gel-EV: n=86, Gel-MSC: n=44, MN-EV: n=30, MN-MSC: n=14 Trials for each groups.

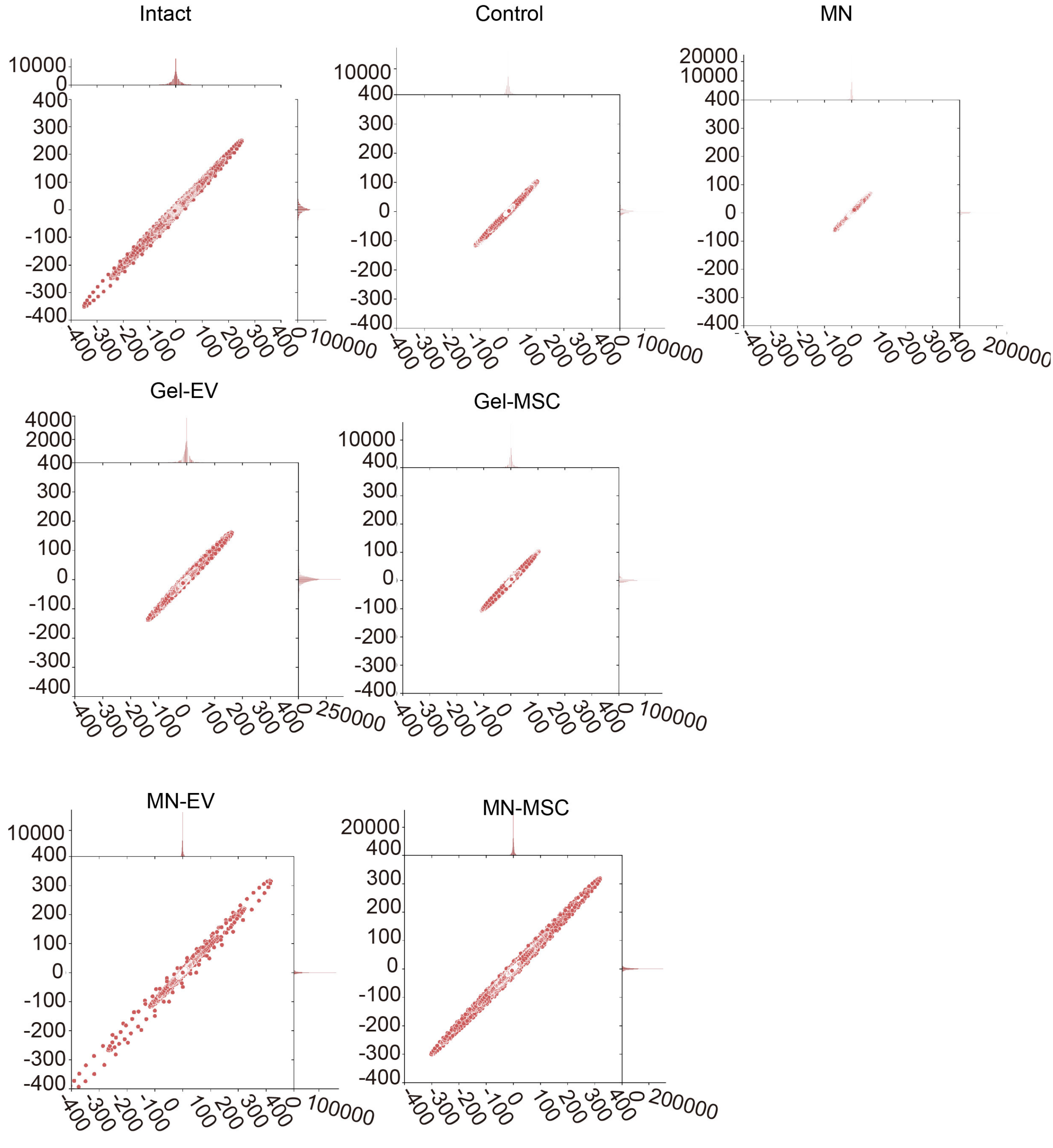

Fig. S11| Related to Fig. 7. Poincaré statistical analysis of the EMG signal amplitude rhythm of GS muscles from rats in the intact, control, MN, Gel-EV, Gel-MSC, MN-EV and MN-MSC groups.

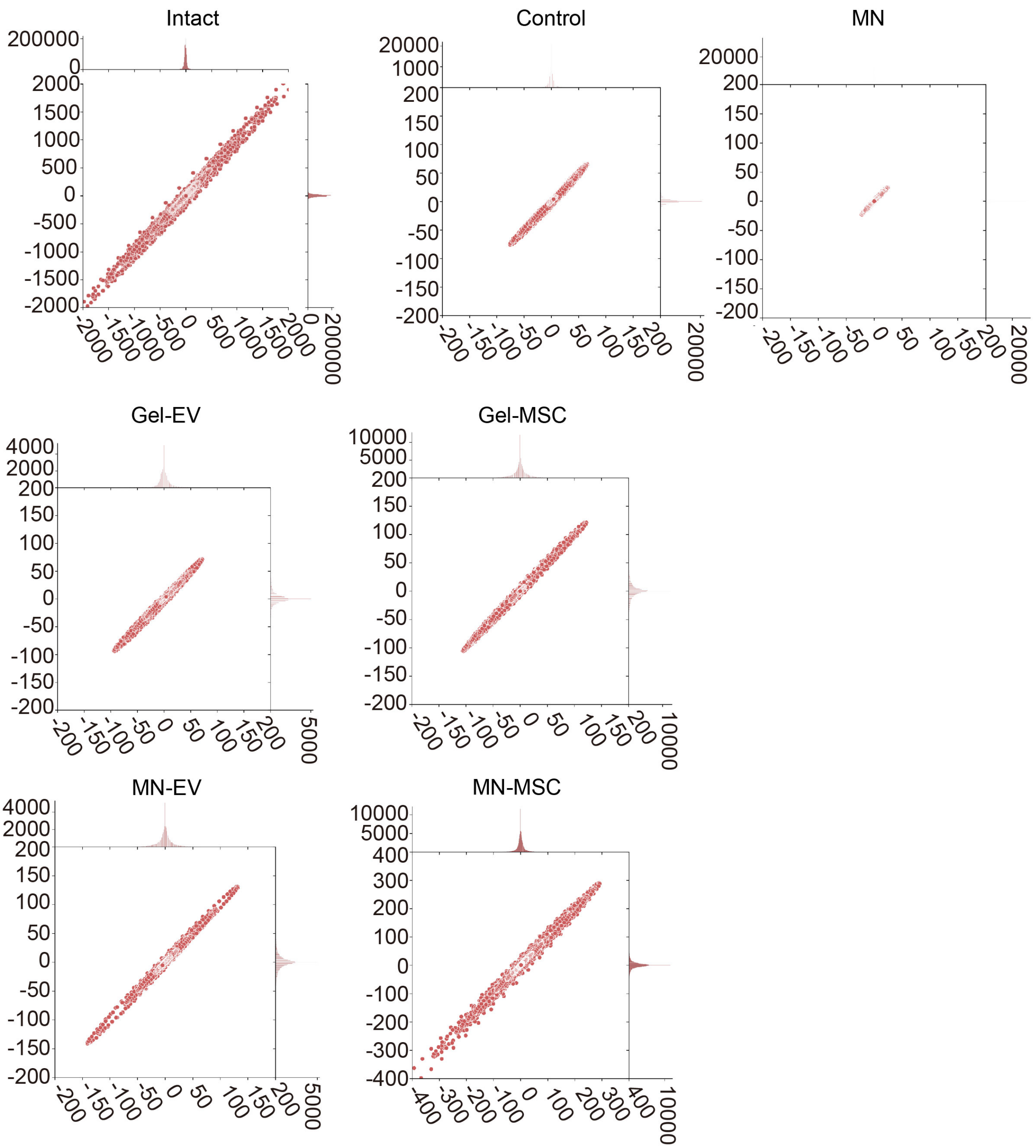

Fig. S12| Related to Fig. 7. Poincaré statistical analysis of the EMG signal amplitude rhythm of TA muscles from rats in the intact, control , MN, Gel-EV, Gel-MSC, MN-EV and MN-MSC groups.

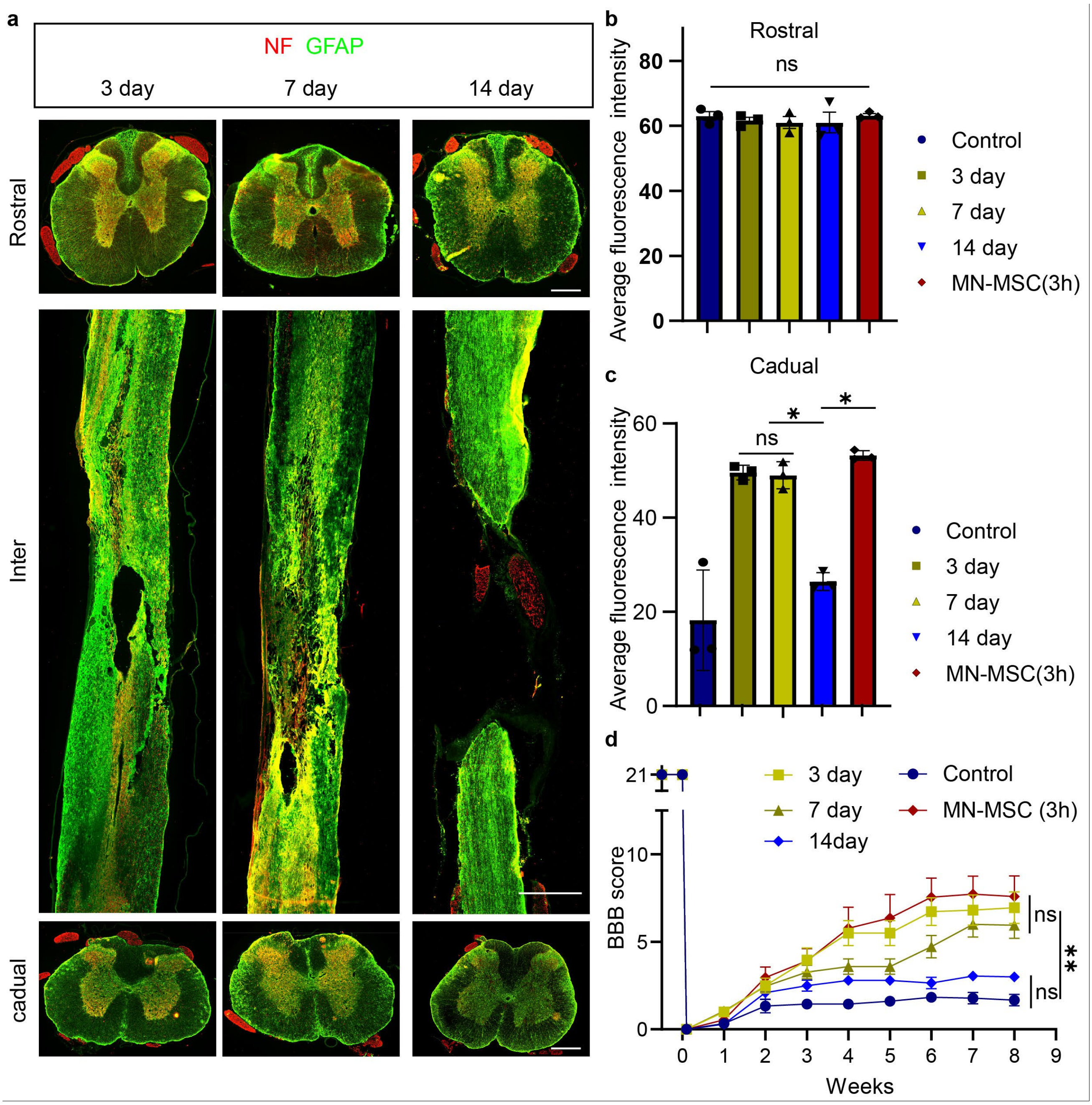

Fig. S13| Detailed analysis of MN-MSC at different implanted time points on the SCI treatment. **a** Representative images of the spinal cord sections stained with GFAP (green) and NF (red) of rats in the three groups (implantation at 3 days, 7 days, and 14 days). Scale bars of rostral and caudal indicate 500 μm, and inter indicate 1 mm. **b-c** Quantification of the average fluorescence intensity of NF immunoreactivity on the rostral (b) and caudal (c) sides of the three groups. Data are shown as the mean ± SEM. Statistical analysis was performed using one-way ANOVA followed by Tukey's multiple comparisons test and two-tailed paired t-tests were used for comparisons between two groups. n = 3 animals chosen randomly of each group. ANOVA for rostral: Total: F= 0.3387, p= 0.8459. ANOVA for caudal: Total: F= 29.07, p <0.0001. 7 days vs 14 days: p=0.0021, 14 days vs MN-MSC(3h): p=0.0005. *p < 0.05, **p < 0.01, ***p < 0.001.**d** The weekly BBB score comparison among the rats in the six groups (implantation at 3 days, 7 days, and 14 days) at 8 weeks after SCI. Data are shown as the mean ± SEM. Two-way ANOVA with Tukey's post-hoc test was used for comparisons among multiple groups, and two-tailed paired t-tests were used for comparisons between two groups. Control: n=9, 3 days: n=11, 7 days: n=11, 14 days: n=10, MN-MSC (3h): n=11animals from each groups. Total: F= 3.959, p<0.0001, 7 days vs 14days, p= 0.0015. *p<0.05 and **p<0.01.

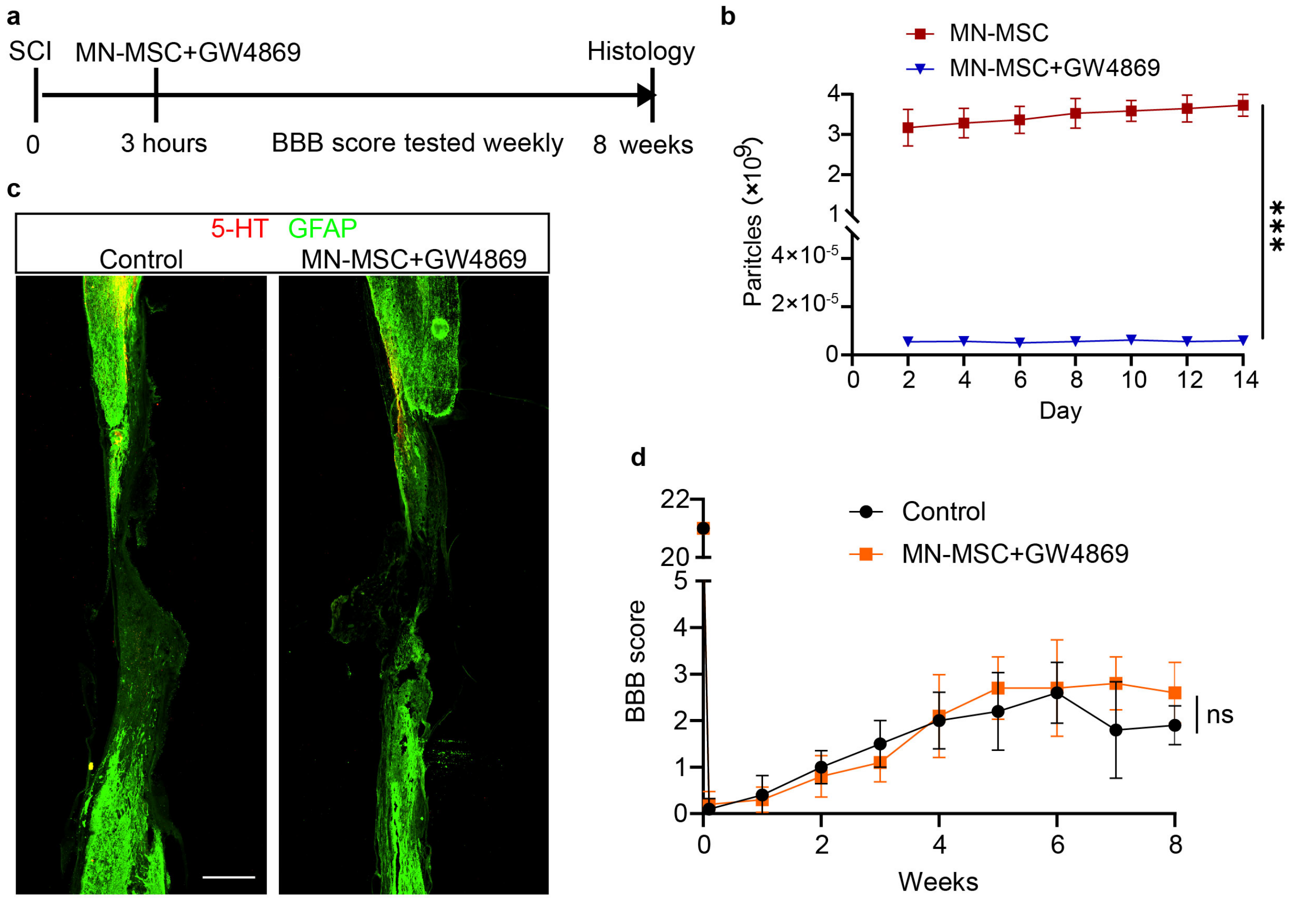

Fig. S14| The effect of MN-MSC without releasing EVs on SCI treatment a Schematic illustration of the experiment. **b** Release profiles of EVs from Transwell experiments. Data are presented as mean ± SEM. Two-tailed paired t-tests were used for comparisons between two groups. n=5 animals of each group. P<0.0001 n = 3 independent samples. **c** Representative images of spinal sections stained with GFAP (green) and 5-HT (red) of rats in two groups (Control, MN-MSC + GW4869(EVs inhibitor)). Scale bar indicate 1 mm. **d** Weekly BBB score comparison among rats in two groups (Control, MN-MSC + GW4869(EVs inhibitor)) at 8 weeks after SCI. Data are shown as the mean ± SEM. Two-tailed paired t-tests were used for comparisons between two groups. n=5 animals of each group. F= 1.334, P= 0.2253, *p<0.05 and **p<0.01.

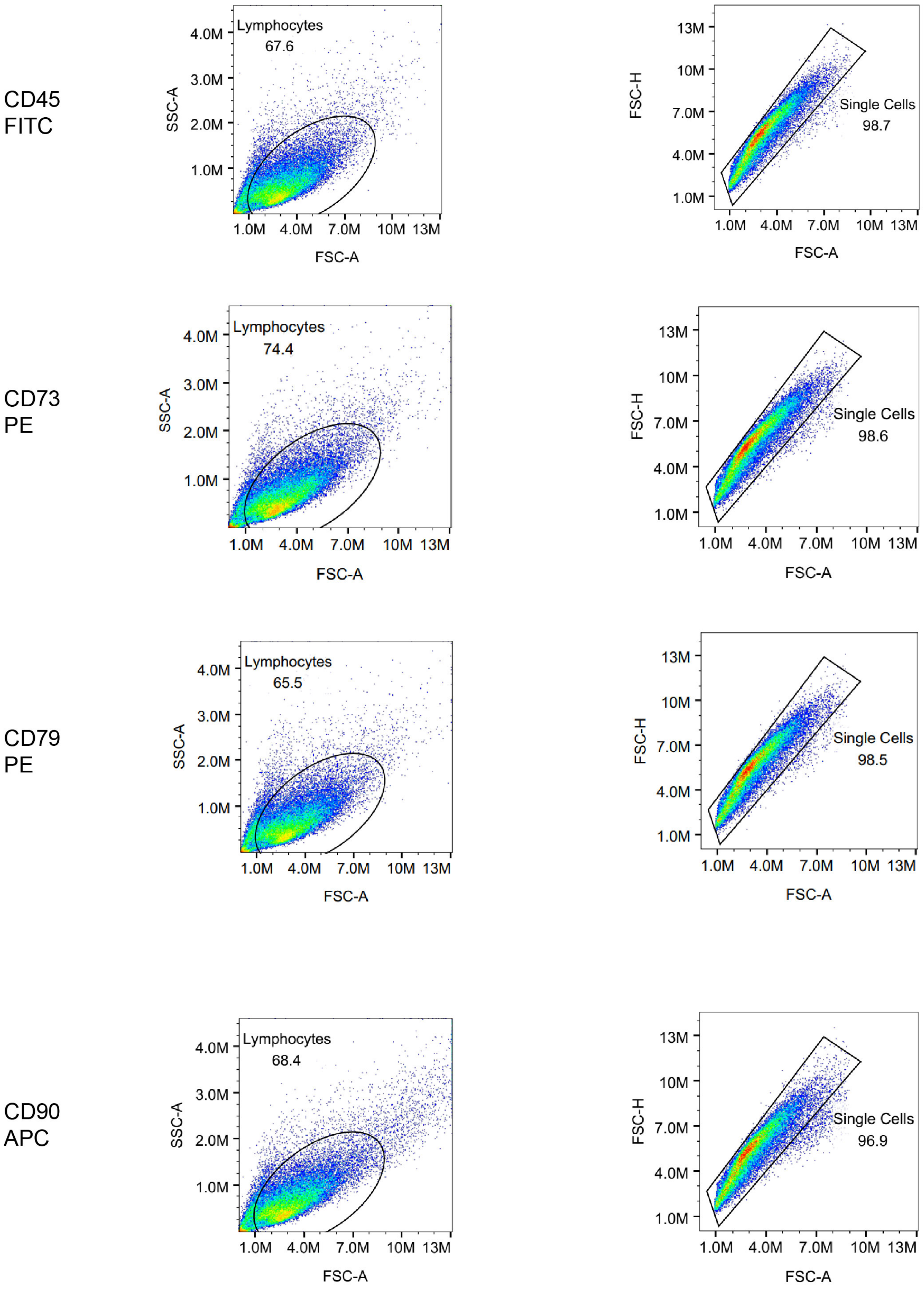

Fig. S15| The flow cytometric gating strategy for cell line with CD45, CD73, CD79 and CD90 antibody.

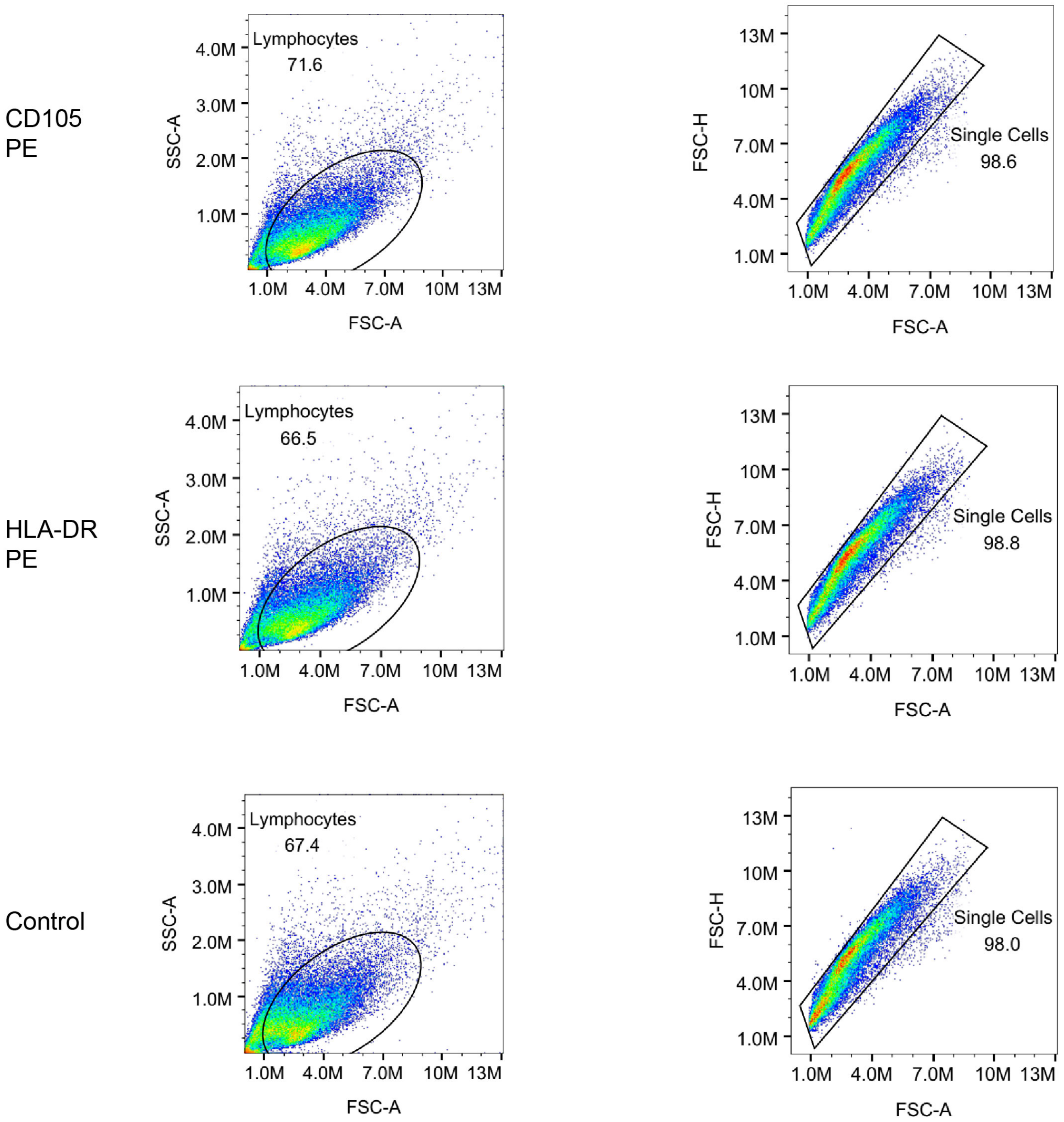

Fig. S16| The flow cytometric gating strategy for cell line with CD105, HLA-DR, Control (without) antibody.

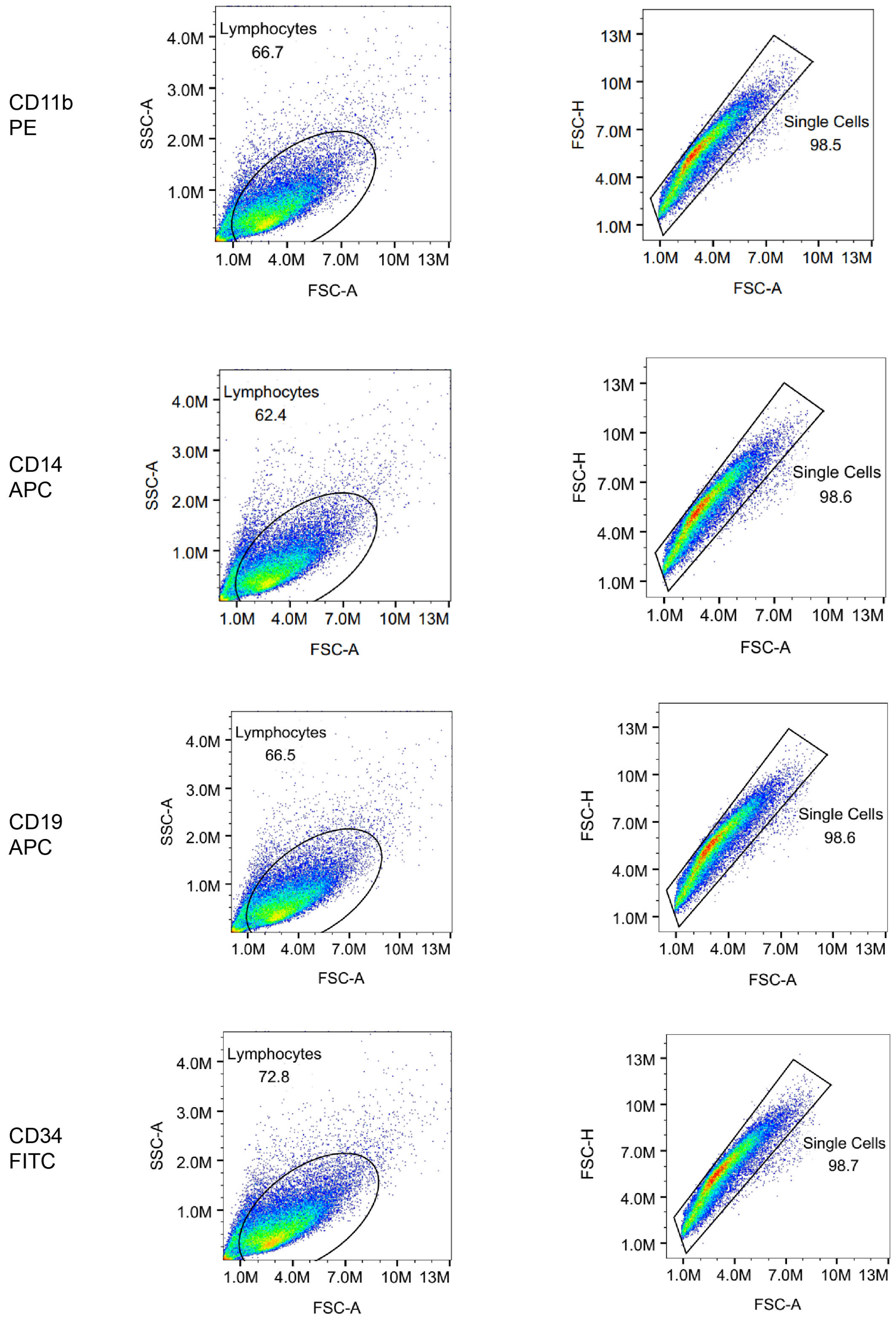

Fig. S17| The flow cytometric gating strategy for cell line with CD11b, CD14, CD19 and CD34 antibody.
