# Supplementary material for: Porous microneedle patch with sustained delivery of extracellular vesicles mitigates severe spinal cord injury
Source: Nat Commun. 2023 Jul 7;14:4011. doi: 10.1038/s41467-023-39745-2 (PMC10328956; doi:10.1038/s41467-023-39745-2)
Supplement: Supplementary file 2 — Description of additional supplementary files [file 41467_2023_39745_MOESM2_ESM.pdf]

<u>Description of additional supplementary files</u>

Supplementary Movie 1 : The movements of the hindlimbs of rats under six treatment groups. The movie shows the hindlimb locomotor joint of the control rats and rats treated by MN, Gel-EV, Gel-MSC, MNEV and MN-MSC 8 weeks after spinal cord injury.
