# Supplementary material for: Porous microneedle patch with sustained delivery of extracellular vesicles mitigates severe spinal cord injury
Source: Nat Commun. 2023 Jul 7;14:4011. doi: 10.1038/s41467-023-39745-2 (PMC10328956; doi:10.1038/s41467-023-39745-2)
Supplement: Supplementary file 4 — Reporting Summary [file 41467_2023_39745_MOESM4_ESM.pdf]

nature portfolio

Corresponding author(s): Xuhua Wang

Last updated by author(s): Jun 16, 2023

Reporting Summary

Nature Portfolio wishes to improve the reproducibility of the work that we publish. This form provides structure for consistency and transparency in reporting. For further information on Nature Portfolio policies, see our Editorial Policies and the Editorial Policy Checklist.

Statistics

For all statistical analyses, confirm that the following items are present in the figure legend, table legend, main text, or Methods section.

n/a	Confirmed	
☐	☒	The exact sample size (*n*) for each experimental group/condition, given as a discrete number and unit of measurement
☐	☒	A statement on whether measurements were taken from distinct samples or whether the same sample was measured repeatedly
☐	☒	The statistical test(s) used AND whether they are one- or two-sided<br>*Only common tests should be described solely by name; describe more complex techniques in the Methods section.*
☒	☐	A description of all covariates tested
☐	☒	A description of any assumptions or corrections, such as tests of normality and adjustment for multiple comparisons
☐	☒	A full description of the statistical parameters including central tendency (e.g. means) or other basic estimates (e.g. regression coefficient) AND variation (e.g. standard deviation) or associated estimates of uncertainty (e.g. confidence intervals)
☐	☒	For null hypothesis testing, the test statistic (e.g. *F*, *t*, *r*) with confidence intervals, effect sizes, degrees of freedom and *P* value noted<br>*Give P values as exact values whenever suitable.*
☒	☐	For Bayesian analysis, information on the choice of priors and Markov chain Monte Carlo settings
☒	☐	For hierarchical and complex designs, identification of the appropriate level for tests and full reporting of outcomes
☒	☐	Estimates of effect sizes (e.g. Cohen's *d*, Pearson's *r*), indicating how they were calculated

Our web collection on statistics for biologists contains articles on many of the points above.

Software and code

Policy information about availability of computer code

Data collection	Transmission electron microscope (TEM)(JEM-1400flash JEOL, JEOL, Japan)<br>Scanning electron microscope (SEM) (Nova Nano 450,Thero FEI, USA)<br>Confocal laser scanning microscopy (AlTi, Nikon, Japan)<br>Virtual digital slice scanning system (VS200, Olympus, Japan)<br>MotoRater (Vicon Motion Systems, UK)<br>ChemiDoc Touch Imaging System(Bio-Rad, USA)<br>microplate reader (iD5, Molecular Devices, USA)<br>universal testing machine (CMT52O5, MTS, China)<br>nanoparticle tracking analysis (Marvin, UK, Nanosight NS500)<br>flow cytometry (ACEA Biosciences, ACEA NovoCyteTM, USA)
Data analysis	For statistics, data were analysed using Graph Prism 8 (8.3.0,GraphPad Software, Inc., USA),Python SciPy(V1.4.1). For microscopy, images were analysed using Fiji(Windows 64,v1.51,NIH). Electromyography (EMG) data analysis and behavioral assessment were using MATLAB (v2020b). Flow cytometry analysis were using CytExpert2.5 Flowjo v10.7.1.

For manuscripts utilizing custom algorithms or software that are central to the research but not yet described in published literature, software must be made available to editors and reviewers. We strongly encourage code deposition in a community repository (e.g. GitHub). See the Nature Portfolio guidelines for submitting code & software for further information.

Data

Policy information about availability of data

All manuscripts must include a data availability statement. This statement should provide the following information, where applicable:

- Accession codes, unique identifiers, or web links for publicly available datasets
- A description of any restrictions on data availability
- For clinical datasets or third party data, please ensure that the statement adheres to our policy

All data generated or analysed during this study are available in this published article and its supplementary information files or from the corresponding author.

Research involving human participants, their data, or biological material

Policy information about studies with human participants or human data. See also policy information about sex, gender (identity/presentation), and sexual orientation and race, ethnicity and racism.

Reporting on sex and gender	*Use the terms sex (biological attribute) and gender (shaped by social and cultural circumstances) carefully in order to avoid confusing both terms. Indicate if findings apply to only one sex or gender; describe whether sex and gender were considered in study design; whether sex and/or gender was determined based on self-reporting or assigned and methods used.* *Provide in the source data disaggregated sex and gender data, where this information has been collected, and if consent has been obtained for sharing of individual-level data; provide overall numbers in this Reporting Summary. Please state if this information has not been collected.* *Report sex- and gender-based analyses where performed, justify reasons for lack of sex- and gender-based analysis.*
Reporting on race, ethnicity, or other socially relevant groupings	*Please specify the socially constructed or socially relevant categorization variable(s) used in your manuscript and explain why they were used. Please note that such variables should not be used as proxies for other socially constructed/relevant variables (for example, race or ethnicity should not be used as a proxy for socioeconomic status).* *Provide clear definitions of the relevant terms used, how they were provided (by the participants/respondents, the researchers, or third parties), and the method(s) used to classify people into the different categories (e.g. self-report, census or administrative data, social media data, etc.)* *Please provide details about how you controlled for confounding variables in your analyses.*
Population characteristics	*Describe the covariate-relevant population characteristics of the human research participants (e.g. age, genotypic information, past and current diagnosis and treatment categories). If you filled out the behavioural & social sciences study design questions and have nothing to add here, write "See above."*
Recruitment	*Describe how participants were recruited. Outline any potential self-selection bias or other biases that may be present and how these are likely to impact results.*
Ethics oversight	*Identify the organization(s) that approved the study protocol.*

Note that full information on the approval of the study protocol must also be provided in the manuscript.

Field-specific reporting

Please select the one below that is the best fit for your research. If you are not sure, read the appropriate sections before making your selection.

☒ Life sciences ☐ Behavioural & social sciences ☐ Ecological, evolutionary & environmental sciences

For a reference copy of the document with all sections, see nature.com/documents/nr-reporting-summary-flat.pdf

Life sciences study design

All studies must disclose on these points even when the disclosure is negative.

Sample size	No statistical method was used to determine sample size. The sample sizes for experiments on cells and animals were determined from our previously published investigations (Ye, J. et al. Rationally Designed, Self-Assembling, Multifunctional Hydrogel Depot Repairs Severe Spinal Cord Injury. Adv Healthc Mater, e2100242) and was sufficient to yield statistical significance.
Data exclusions	No data was excluded
Replication	All experiments were repeated three times as indicated in the figure legends, and the experimental results were reproducible.
Randomization	For cell-based assays, cultured cells were randomly allocated to different experimental groups. For in vivo experiments, rats were randomly assigned to each experimental group before treatment
Blinding	For in vivo studies, experimenters were blinded to the group allocation. Blinding was performed when conducting quantitative analysis for immunohistochemical, bioluminescence imaging and quantification, in vivo fluorescence quantification and immunofluorescence. Behavior tests (BBB score) were determined from independent persons who were unaware of the expected therapeutic outcome. For in vitro

studies, all the experiments were conducted following double-blind manner.

Reporting for specific materials, systems and methods

We require information from authors about some types of materials, experimental systems and methods used in many studies. Here, indicate whether each material, system or method listed is relevant to your study. If you are not sure if a list item applies to your research, read the appropriate section before selecting a response.

Materials & experimental systems

n/a	Involved in the study
	☒ Antibodies
	☒ Eukaryotic cell lines
☒	☐ Palaeontology and archaeology
	☒ Animals and other organisms
☒	☐ Clinical data
☒	☐ Dual use research of concern
☒	☐ Plants

Methods

n/a	Involved in the study
☒	☐ ChIP-seq
	☒ Flow cytometry
☒	☐ MRI-based neuroimaging

Antibodies

Antibodies used

Chicken anti-GFAP [abcam (ab134436, 1:500)]
GAPDH (Human Specific) Rabbit mAb [Abclonal (AC036), 1:500]
rabbit anti-neurofilament (NF) heavy polypeptide [abcam (ab8135), 1:500]
goat anti 5-HT antibody [abcam (ab66047), 1:500]
rabbit anti-NeuN [abcam (ab177487), 1:1000]
Rabbit polyclonal to RFP[abcam (ab62341), 1:500]
rabbit anti-MBP [abcam (ab218011), 1:500]
goat anti-IBA1 [abcam(ab5076), 1:500]
rabbit anti-CD31 [R&Drd system (AF3628), 1:500]
GAPDH Rabbit Monoclonal Antibody [Beyotime (AF1186,1:1000)]
rabbit anti-TGF- β[Abclonal(A2124), 1:1000]
rabbit anti-Bax [Abclonal (A0207),1:1000]
rabbit anti-MMP9 [Abclonal (A0289) ,1:1000]
rabbit anti-Arginase 2 (ARG2) [Abclonal (A19233) ,1:1000]
Donkey anti-Chicken IgY H&L (FITC) [Abcam (ab63507), 1:500]
Donkey anti-Rabbit IgG H&L (Alexa Fluor® 555) [Abcam (ab150062), 1:500]
Rabbit anti-Goat IgG H&L (Alexa Fluor® 555) [Abcam (ab150142), 1:500]
Donkey anti-Rabbit secondary antibodies (HRP) [beyotime (A0208), 1:1000]
Rabbit anti-iNOS [proteintech (80517-1-RR), 1:2000]
Rabbit anti-TNF-α [proteintech (17590-1-AP), 1:1000]
Rabbit anti-IL-1β [Abclonal (A20527), 1:1000]
Rabbit α-Tubulin [beyotime (AG0126), 1:1000]
PE Mouse anti-Human CD105 [BD Pharmingen™, 560839, 1:20],
APC Mouse Anti-Human CD90 [BD Pharmingen™, 561971, 1:20],
PE Mouse Anti-Human CD73 [BD Pharmingen™, 550257, 1:20],
FITC Mouse Anti-Human CD45 [BD Pharmingen™, 561865, 1:20],
FITC Mouse Anti-Human CD34 [BD Pharmingen™, 560942, 1:20],
APC Mouse Anti-Human CD19 [BD Pharmingen™, 561742, 1:20],
APC Mouse Anti-Human CD14 [BD Pharmingen™, 555399, 1:20],
PE Mouse Anti-Human CD11b [BD Pharmingen™, 555388, 1:20],
PE Mouse Anti-Human CD79a [BD Pharmingen™, 561942, 1:20],
PE Mouse Anti-Human HLA-DR [BD Pharmingen™, 555812, 1:20].

Validation

Anti-GFAP [abcam (ab134436, 1:500)],species:chicken, reacts with: rat, suitable for ：icc/if. Lan G et al. Astrocytic VEGFA: An essential mediator in blood-brain-barrier disruption in Parkinson's disease. Glia 70:337-353 (2022).

GAPDH (Human Specific) Rabbit mAb [Abclonal (AC036), 1:500], ,species:rabbit, reacts with: Human, suitable for ：WB, icc/if. USP22 positively modulates ERα action via its deubiquitinase activity in breast cancer. Published:2020, Journal:Cell Death Differ.

Anti-neurofilament (NF) heavy polypeptide [abcam (ab8135), 1:500], species: rabbit, reacts with:Mouse, Rat. Suitable for: IHC-FrFl, WB.Li H et al. Local continuous glial cell derived neurotrophic factor release using osmotic pump promotes parasympathetic nerve rehabilitation in an animal model of cavernous nerve injury induced erectile dysfunction. Transl Androl Urol 10:258-271 (2021).

Anti 5-HT antibody [abcam (ab66047), 1:500], species: goat, Reacts with: Species independent. Suitable for: IHC-FoFr .Widmayer P et al. Short-term high fat feeding induces inflammatory responses of tuft cells and mucosal barrier cells in the murine stomach. Histol

Histopathol 38:273-286 (2023).

Anti-NeuN [abcam (ab177487), 1:1000], species: rabbit, reacts with: Mouse, Rat, Sheep, Goat, Cat, Dog, Human, Zebrafish, Common marmoset. Suitable for: Flow Cyt (Intra), IHC (PFA fixed), mIHC, IHC-P, WB, ICC/IF, IHC-Fr. Qiao X et al. Dual-specificity phosphatase 15 (DUSP15) in the nucleus accumbens is a novel negative regulator of morphine-associated contextual memory. Addict Biol 26:e12884 (2021).

Anti-RFP[abcam (ab62341), 1:500], species: rabbit, Reacts with: Recombinant fragment, Suitable for: WB, ICC/IF, IHC-Fr, IHC-P, IP, IHC-FrFl. Zhang L et al. S100A11 Promotes Liver Steatosis via FOXO1-Mediated Autophagy and Lipogenesis. Cell Mol Gastroenterol Hepatol 11:697-724 (2021).

Anti-MBP [abcam (ab218011), 1:500], species: rabbit, Reacts with: Mouse, Rat, Human, Suitable for: WB, IHC-P, IHC-Fr. He X et al. BAY61-3606 attenuates neuroinflammation and neurofunctional damage by inhibiting microglial Mincle/Syk signaling response after traumatic brain injury. Int J Mol Med 49:N/A (2022).

Anti-IBA1 [abcam(ab5076), 1:500], species: goat, Reacts with: Rat, Human, Suitable for: IHC-P, WB. Keane L et al. mTOR-dependent translation amplifies microglia priming in aging mice. J Clin Invest 131:N/A (2021).

Anti-CD31 [R&Drd system (AF3628), 1:500], species: rabbit, Reacts with: Mouse, Rat. Suitable for: WB, Flow Cytometry, Immunohistochemistry, CyTOF-ready, Immunocytochemistry. gelation for first-aid wound treatment. Authors: Q Zhu, X Zhou, Y Zhang, D Ye, K Yu, W Cao, L Zhang, H Zheng, Z Sun, C Guo, X Hong, Y Zhu, Y Zhang, Y Xiao, TG Valencak, T Ren, D Ren. Biomaterials research, 2023;27(1):6.

GAPDH Monoclonal Antibody [Beyotime (AF1186,1:1000)], species: rabbit, Reacts with: H, Human; M, Mouse; R, Rat; C, Chicken; Mk, Monkey; Z, Zebrafish. Suitable for: WB, IP, IF, IHC, ICC, FC, 1. Jinchao Jia, Ferritin triggers neutrophil extracellular trap-mediated cytokine storm through Msr1 contributing to adult-onset Still's disease pathogenesis. Nat Commun. 2022 Nov 10;13(1):6804.;doi: 10.1038/s41467-022-34560-7. (IF 12.121)

Anti-TGF-β [Abclonal(A2124), 1:1000], species: rabbit, Reacts with: Human,Mouse,Rat, Suitable for: WB, IHC, IF, ICH, ELISA , Clusterin negatively modulates mechanical stress-mediated ligamentum flavum hypertrophy through TGF-β1 signaling

Anti-Bax [Abclonal (A0207),1:1000], species: rabbit, Reacts with: Human, Mouse, Rat. Suitable for: WB, IHC-P, IF/ICC, IP, The lncRNA MACC1-AS1 promotes gastric cancer cell metabolic plasticity via AMPK/Lin28 mediated mRNA stability of MACC1.

Anti-MMP9 [Abclonal (A0289) ,1:1000], species: rabbit, Reacts with: Mouse, Rat, Suitable for: IHC-P, IF/ICC. miR-301a promotes lung tumorigenesis by suppressing Runx3.

Anti-Arginase 2 (ARG2) [Abclonal (A19233) ,1:1000], species: rabbit, Reacts with: Human. Suitable for: WB, IHC-P.

Anti-iNOS [proteintech (80517-1-RR), 1:2000], species: rabbit, Reacts with: Human, Mouse, Rat. Suitable for: IHC, WB, ELISA.
Anti-TNF-α [proteintech (17590-1-AP), 1:1000], species: rabbit, Reacts with: Human, Mouse, Suitable for: WB, ELISA, Macrophage Inactivation by Small Molecule Wedelolactone via Targeting sEH for the Treatment of LPS-Induced Acute Lung Injury. Authors - Juan Zhang

Anti-IL-1β [Abclonal (A20527), 1:1000], species: rabbit, Reacts with: Human, Mouse, Rat, Suitable for: WB, IHC-P.

Tubulin [beyotime (AG0126), 1:1000], species: rabbit, Reacts with: H, Human; M, Mouse; R, Rat; Suitable for: WB, IHC-P, IF/ICC, FC.

PE Mouse anti-Human CD105 [BD Pharmingen™, 560839, 1:20], APC Mouse Anti-Human CD90 [BD Pharmingen™, 561971, 1:20], PE Mouse Anti-Human CD73 [BD Pharmingen™, 550257, 1:20], FITC Mouse Anti-Human CD45 [BD Pharmingen™, 561865, 1:20], FITC Mouse Anti-Human CD34 [BD Pharmingen™, 560942, 1:20], APC Mouse Anti-Human CD19 [BD Pharmingen™, 561742, 1:20], APC Mouse Anti-Human CD14 [BD Pharmingen™, 555399, 1:20], PE Mouse Anti-Human CD11b [BD Pharmingen™, 555388, 1:20], PE Mouse Anti-Human CD79a [BD Pharmingen™, 561942, 1:20], PE Mouse Anti-Human HLA-DR [BD Pharmingen™, 555812, 1:20]. Pawelec G, Ziegler A, Wernet P. Dissection of human allostimulatory determinants with cloned T cells: stimulation inhibition by monoclonal antibodies TU22, 34, 35, 36, 37, 39, 43, and 58 against distinct human MHC class II molecules. Hum Immunol. 1985; 12 (3):165-176. (Biology).

Eukaryotic cell lines

Policy information about cell lines and Sex and Gender in Research

Cell line source(s)	Human embryonic stem cell (ESC)-derived MSCs were generated by Ysbiotech, Hangzhou, China (YS™ hESC-MSC) and cultured at 37°C in a humidified atmosphere of 5% CO2 in serum-free medium for MSCs (NC0103+NC0103. S, Yocon, Beijing, China) as previously reported(X. Jiang et al, Stem Cell Res Ther 13, 313,2022). The H9 human embryonic stem cell line (WA09), originally generated by the National Stem Cell Bank c/o WiCell Research Institute (USA), was obtained from the stem cell bank at the Institute of Biochemistry and Cell Biology, CAS.
Authentication	MSC cells were authenticated by Flow cytometry analysis. H9 human embryonic stem cells were authenticated by genomic sequencing.

Mycoplasma contamination	All cell lines used in this study were tested negative for mycoplasma contamination.
Commonly misidentified lines (See ICLAC register)	No commonly misidentified cell lines were used

Animals and other research organisms

Policy information about studies involving animals; ARRIVE guidelines recommended for reporting animal research, and Sex and Gender in Research

Laboratory animals	Sprague—Dawley rats (about 8 weeks, 220-250 g) were purchased from the Experimental Animal Center of the Zhejiang Academy of Medical Science, Hangzhou, China. All animals were housed in a specific pathogen-free environment at ambient temperature (24 ± 2 ℃), air humidity 40- 70% and 12 h dark/12 h light cycle.
Wild animals	No wild animals were used in this study.
Reporting on sex	According to previous experimental menthod， Female mice were used in all experiments.
Field-collected samples	This study did not involve any sample collected from the field.
Ethics oversight	All animal experiments ethical approval were approved by the Institutional Animal Care and used following the provisions of Zhejiang University Animal Experimentation Committee and Independent Ethics Committee (ZJU202010110).

Note that full information on the approval of the study protocol must also be provided in the manuscript.

Flow Cytometry

Plots

Confirm that:

- [x] The axis labels state the marker and fluorochrome used (e.g. CD4-FITC).
- [x] The axis scales are clearly visible. Include numbers along axes only for bottom left plot of group (a 'group' is an analysis of identical markers).
- [x] All plots are contour plots with outliers or pseudocolor plots.
- [x] A numerical value for number of cells or percentage (with statistics) is provided.

Methodology

Sample preparation	Human embryonic stem cell (ESC)-derived mesenchymal stem cells (MSCs) were generated by Ysbiotech, Hangzhou, China (YSTM hESC-MSC) and cultured at 37°C in a humidified 5% CO2 atmosphere in serum-free medium for MSCs (NC0103 +NC0103. S, Yocon, Beijing, China), as previously reported with some modifications (X. Jiang et al, Stem Cell Res Ther 13, 313, 2022). The H9 human embryonic stem cell line, originally generated by the National Stem Cell Bank c/o WiCell Research Institute (USA), was obtained from the stem cell bank at the Institute of Biochemistry and Cell Biology, CAS. ESC-MSCs were differentiated from ESCs as follows. Briefly, H9-ESC colonies were dissociated into small clumps using TrypLE Express and cultured in ultralow-attachment plates with E8 medium (Gibco, Grand Island, NY, USA). After 1 week, embryoid bodies were harvested and cultured in MSC induction medium (high-glucose Dulbecco's modified Eagle's medium, 10% fetal bovine serum, and 1 mM L-glutamine). After 15 days, embryoid body outgrowths were sub-cultured using TrypLE Express, yielding passage 0 MSCs. MSCs were then cultured in serum-free medium. When proliferating colonies reached near confluence, hESC-MSCs were passaged using stem cell moderate digestive enzymes (NC1004, Yocon, Beijing, China). After 4 passages, MSCs were analyzed by flow cytometry. After digestion and centrifugation, cells were diluted in PBS, counted, and 5 x 105 cells were added to each tube. Cells were centrifuged at 300 x g for 5 min and resuspended in PBS. Then, 5 L of antibody was added to each tube to a total volume of 100 L. Tubes were vortexed and incubated at room temperature in the dark for 15 min. PBS (1 mL) was added to each tube and tubes were centrifuged at 300 x g for 10 min. The supernatant was discarded and cells were resuspended in 500 L PBS. Cells were then analyzed by flow cytometry.
Instrument	Beckman cytoflex LX
Software	CytExpert2.5 Flowjo v10.7.1
Cell population abundance	N/A no sorting was performed
Gating strategy	Use unlabeled cells as blank controls and detect them on the instrument. Set the position of the positive gate according to the background fluorescence of the unlabeled cells. Run the labeled sample tubes under the same acquisition conditions to obtain the percentage of expression of specific antigens.

- [x] Tick this box to confirm that a figure exemplifying the gating strategy is provided in the Supplementary Information.
